# The *Mummy Explorer*—a self-regulated open-access online teaching tool

**DOI:** 10.1093/emph/eoad009

**Published:** 2023-04-27

**Authors:** Anja Furtwängler, Chris Baumann, Kerttu Majander, Shevan Wilkin, Nadja Tomoum, Frank Rühli, Adrian V Jaeggi, Patrick Eppenberger, Nicole Bender, Verena J Schuenemann

**Affiliations:** Institute for Evolutionary Medicine, University of Zurich, Winterthurerstrasse 190, 8057 Zurich, Switzerland; Faculty of Science, University of Helsinki, Gustaf Hällströmin katu 2, 00014, Finland; Biogeology, Department of Geosciences, University of Tübingen, Hölderlinstraße 12, 72074 Tübingen, Germany; Institute for Evolutionary Medicine, University of Zurich, Winterthurerstrasse 190, 8057 Zurich, Switzerland; Department of Evolutionary Anthropology, University of Vienna, 1030 Vienna, Austria; Institute for Evolutionary Medicine, University of Zurich, Winterthurerstrasse 190, 8057 Zurich, Switzerland; Australian Research Centre for Human Evolution (ARCHE), Griffith University, Brisbane 4111, Australia; Max Planck Institute for Geoanthropology, 07745 Jena, Germany; Institute for Evolutionary Medicine, University of Zurich, Winterthurerstrasse 190, 8057 Zurich, Switzerland; Institute for Evolutionary Medicine, University of Zurich, Winterthurerstrasse 190, 8057 Zurich, Switzerland; Institute for Evolutionary Medicine, University of Zurich, Winterthurerstrasse 190, 8057 Zurich, Switzerland; Institute for Evolutionary Medicine, University of Zurich, Winterthurerstrasse 190, 8057 Zurich, Switzerland; Institute for Evolutionary Medicine, University of Zurich, Winterthurerstrasse 190, 8057 Zurich, Switzerland; Institute for Evolutionary Medicine, University of Zurich, Winterthurerstrasse 190, 8057 Zurich, Switzerland; Department of Evolutionary Anthropology, University of Vienna, 1030 Vienna, Austria; Human Evolution and Archaeological Sciences (HEAS), University of Vienna, 1030 Vienna, Austria

## Abstract

**Background and objectives:**

Virtual teaching tools have gained increasing importance in recent years. In particular, the COVID-19 pandemic has reinforced the need for media-based and self-regulated tools. What is missing are tools that allow us to interlink highly interdisciplinary fields such as evolutionary medicine and, at the same time, allow us to adapt content to different lectures.

**Methodology:**

We designed an interactive online teaching tool, namely, the *Mummy Explorer,* using open-access software (Google Web Designer), and we provided a freely downloadable template. We tested the tool on students and lecturers of evolutionary medicine using questionnaires and improved the tool according to their feedback.

**Results:**

The tool has a modular design and provides an overview of a virtual mummy excavation, including the subfields of palaeopathology, paleoradiology, cultural and ethnographic context, provenance studies, paleogenetics, and physiological analyses. The template allows lecturers to generate their own versions of the tool for any topic of interest by simply changing the text and pictures. Tests undertaken with students of evolutionary medicine showed that the tool was helpful during their studies. Lecturers commented that they appreciated having a similar tool in other fields.

**Conclusions and implications:**

*Mummy Explorer* fills a gap in the virtual teaching landscape of highly interdisciplinary fields such as evolutionary medicine. It will be offered for free download and can be adapted to any educational topic. Translations into German and possibly other languages are in progress.

## BACKGROUND AND OBJECTIVES

The presence and quality of online platforms for data visualization—especially for academic education—have greatly increased in recent years. Since the COVID-19 pandemic, the importance of digital education and online teaching has grown and will probably continue to grow in the future [1]. Online and media-based teaching, in general, can increase teaching effectiveness by improving self-regulated learning and student motivation [2, 3].

Based on such findings, many online teaching tools, websites, and platforms have been created for different disciplines in the life sciences. Several of them allow for students’ interactions and feedback. Some platforms allow lecturers to add didactical elements to their online teaching materials or to create entire ready-to-use courses. Many websites also provide theoretical backgrounds and didactic and educational information for lecturers [1]. While virtual learning increases independent learning skills, blended learning combines on-site and online teaching elements, for instance, with the aid of different media [4, 5]. This vast array of didactical tools offers lecturers the most appropriate approach for each educational situation.

However, one common problem in online lecture series is the absence of a systematic and logical connection among the varied contents of individual lectures. This is especially true for strongly interdisciplinary fields such as evolutionary medicine. Evolutionary medicine is an emerging research and teaching field in which evolutionary biological principles are applied to medical topics with the goal of better understanding health and human susceptibility to diseases [6]. Education in evolutionary medicine at the university level is slowly increasing in the USA and Europe. However, it is mostly taught in anthropology or biology departments and is still rarely taught in medical schools [7]. Especially in medical schools, teaching evolutionary medicine is expected to improve the biological understanding of human health and pathologies, and this knowledge should lead to better-informed medical practice [8, 9]. One challenge is that teachings on evolutionary medicine can cover very different fields, such as genetics and phylogeny, life history theory, or diseases of evolutionary mismatch, even within single lectures [10], which makes it challenging to communicate to students the connections among these subtopics. Another problem of online teaching is that it is difficult for students to understand the exact processes, procedures, and problems of the various practical examination methods of specific subfields, particularly due to the lack of practical laboratory courses, field expeditions, and medical examination experiences. Again, such types of content are often disconnected from theoretical concepts and therefore challenging to interconnect with each other.

Furthermore, while classical textbooks for evolutionary medicine exist [11, 12], as well as several websites (https://isemph.org/EvMedEd) or instructional videos (https://www.youtube.com/user/YaleCourses), there is no specific online teaching tool that can be adapted to different subtopics in this field. Usually, it is not possible to adapt the prespecified contents of common teaching platforms to one’s own teaching topic, which makes it challenging to find a single suitable online tool or makes it necessary to combine several different tools that are not interlinked with each other.

We developed a self-explanatory, innovative online educational tool to overcome these difficulties. This tool allows for a systematic connection of educational contents that otherwise seem abstract and challenging to put into context. In the first version, we created a virtual examination of a mummy and called our teaching tool the *Mummy Explorer*. During a virtual journey through a palaeopathological examination of a mummy, we didactically explain how the logical sequence of the individual examination steps is to be understood and how the collected data can be interpreted in their historical and cultural context. At the same time, appropriate didactical tools, such as images, videos, links, text elements, etc., are added at each step to help visualize the methods and explain them understandably. All subtopics are interlinked with each other to show their connections. This online tool can be used for blended learning as a reference point, a throughline during lectures, and as a virtual self-regulated learning tool for repeating the learned materials online.

This online tool is open access and has a modular structure, meaning its content is easily interchangeable and thus designed for a large target audience. Lecturers can download a template and replace the image and text elements as they wish in a user-friendly way, adapting the tool to their own teaching topic. Instead of the mummy, a skeleton, a living human, a patient, a child, an ape, or even a theoretical concept can be placed in the centre, and several different subfields can be linked to the central element and each other. Therefore, didactical applications in medicine, biology, archaeology, ethnology, and other human/biological sciences are conceivable.

The aim of the present article is, therefore, to present a teaching tool that shall help make connections across subtopics of an interdisciplinary field that might seem to students to be unrelated areas of knowledge. Furthermore, the tool shall allow an individual learning experience for each student to prepare, accompany, or repeat the teaching content in a self-regulated way. For teachers, the tool allows the presentation of their contents in an interlinked and interchangeable way, which can be used in in-person, blended, and virtual learning environments. While interlinked websites of knowledge such as Wikipedia exist, no teaching web tool allows entirely customizable and arbitrarily interchangeable content, simultaneously offering interlinks and external links to additional content.

## METHODOLOGY

### Didactical concepts

The *Mummy Explorer* was designed to demonstrate how single subfields of a specific topic are related to each other, how methodological steps within each subfield are related, how they build on each other, how they work, what insights are generated in each step when to use which method and what limitations the methods have. Every step is explained with text and illustrated with figures. For each method or subtopic, further reading is provided through hyperlinks. A feedback option is provided. The tool allows for a logical alignment of didactical elements during the whole teaching cycle.

The *Mummy Explorer* is designed following established learning theories, such as Bloom’s Taxonomy of Educational Objectives or Gagne’s Nine Events of Instruction, especially with online education theories [13]. For instance, one of the most relevant features of the *Mummy Explorer* addresses one of Siemens’ Eight Principles of Connectivism, that is, the ‘ability to see connections between fields, ideas, and concepts is a core skill’ [14]. According to Terry Anderson, this tool profits from the internet’s hyperlink functions that are comparable with how humans store and access knowledge. Anderson conceived a comprehensive and integrated online didactical model, including structured learning tools and self-paced education models for independent online learning [15]. Self-paced or self-regulated learning is a three-stage process encompassing preparation, performance, and appraisal. Each stage includes the four aspects of cognition, metacognition, motivation, and emotion. Different theoretical models of self-regulated learning exist, with different strengths and performances for different educational levels [16].

The *Mummy Explorer* is suited to substitute for (=virtual learning) and to accompany (=blended learning) existing on-site lecture series. Students can use the *Mummy Explorer* to repeat what they have learned, to better understand the overall context of a lecture and to deepen their grasp of the content. The lecturer can change the *Mummy Explorer’s* content and adapt it to a wide range of educational fields, such as human and veterinary medicine, nursing, evolutionary biology, ecology, archeology, ethnology, and other humanities. This inter- and transdisciplinary approach is a novel contribution of the tool and makes it unique among media-based self-regulated learning tools.

### Software used

The online tool was programmed in HTML5, which runs on all commonly used browsers and enables media data integration. Since one goal of the project was to allow other lecturers to use a template to create their own version of the tool, freely available software (Google Web Designer) was used to generate the webpage. This program also includes a graphical user interface (GUI) that makes it accessible to people without sophisticated knowledge of HTML programming. In combination with a Google account, the program offers an option to publish it on Google drive and to host it via a web service (https://drv.tw/), which requires no further knowledge about hosting websites.

Pages within the tool can be set up with the abovementioned software. All features can be added and manipulated via the GUI. Content can be added in similar ways to many other applications. Furthermore, events can be added as actions if the viewer clicks or hovers over pictures and texts, which makes the webpage interactive. The software also provides the option to use preinstalled or saved templates. This feature can help other lecturers reuse our tool and adapt it to their needs. A version of the tool with placeholders for texts and pictures was saved as a template and can be loaded into the software. Therefore, creating one’s own tool consists of replacing the text elements and swapping the placeholders for pictures (detailed instructions on how to use the template and change contents can be found in the Supplementary Information).

### Survey

Students and lecturers were asked to complete separate versions of a questionnaire to review the tool’s performance. According to their answers and feedback, the online tool or the manual for using the template was adjusted.

The questions for the students (Table 1, Supplementary Information Note II) were designed to first obtain information on how the technical aspects of the tool perform. The questions targeted aspects such as the loading and display of content. The second part of the survey asked if the students found the content helpful and if it was displayed coherently. The tool was tested in two consecutive study years with students at the bachelor’s and master’s levels. In the second year, the questionnaire used was improved in terms of replacing binary responses with a Likert scale. Furthermore, a question about the previewed usefulness of the tool was added. In total, the tool and the survey were shown to 424 students in four courses of the Natural Sciences Faculty and in one course of the Medical Faculty of the University of Zurich.

**Table 1.** Questions and answers of the 2021 survey for the students. The total number of participants in the survey was 67 students in different courses with related content to the online tool

Question Nr.	Question	Number of answers	Nr. Yes	% Yes
***Content-related questions***				
1	Does the tool provide an overview of possible analyses for the study of mummies?	67	66	98,5%
2	Did you get an idea of what data can be collected and for what purpose?	66	65	98.5%
3	Does the tool contain enough pictures and schemes?	65	51	78.5%
4	Did you get an idea of how the different research areas are linked?	64	48	75.0%
5	Are enough references for further reading provided?	63	56	88.9%
6	Is there something you did not understand? Why?	19	N/A	
***Technical questions***				
7	Could you open the tool?	64	59	92.2%
8	Do the pages load fast enough?	64	51	79.7%
9	Did you have any other technical difficulties?	32	N/A	
10	Is the content of the pictures visible and the resolution good enough?	64	62	96.9%
*General Feedback*				
11	Space for more comments. What did you like? What should be improved?	33	N/A	

The questionnaire for the lecturers was constructed in a similar manner (Table 2, Supplementary Information Note III). However, in the lecturers’ version, the technical aspects of changing the template were targeted in more detail. The questions also asked if the provided instruction was understandable and enabled lecturers to set up their own version of the tool. Lecturers asked to evaluate the tool were from the IEM and the natural sciences faculty at the University of Zurich.

**Table 2.** Questions and answers of the survey 2022 for the students. The total number of participants in the survey was 34 students in different courses with related content to the online tool.

Question Nr.	Question					Number of answers	Mean score	% of maximal score
Scale	1	2	3	4	5			
***Content-related questions***								
1	Does the tool provide an overview of possible analyses for the study of mummies?							
*Answers*	*0*	*0*	*1*	*11*	*18*	*30*	*4.56*	*91.2%*
2	Did you get an idea of what data can be collected and for what purpose?							
*Answers*	*0*	*1*	*0*	*8*	*19*	*28*	*4.60*	*92.0%*
3	Does the tool contain enough pictures and schemes?							
*Answers*	*4*	*3*	*3*	*6*	*10*	*26*	*3.58*	*71.6%*
4	Did you get an idea of how the different research areas are linked?							
*Answers*	*0*	*2*	*3*	*12*	*11*	*28*	*4.14*	*82.8%*
5	Are enough references for further reading provided?							
*Answers*	*4*	*2*	*1*	*10*	*11*	*28*	*3.79*	*75.8%*
6	Is there something you did not understand?							
Scale	Yes		No					
*Answers*	*2*		*20*			*22*		*90.0%*
7	Do you think the Mummy Explorer can support your learning experience?							
*Answers*	*3*	*2*	*0*	*7*	*11*	*23*	*3.91*	*78.2%*
***Technical questions***								
7	Could you open the tool?							
*Answers*	*0*	*0*	*0*	*3*	*31*	*34*	*4.91*	*98.2%*
8	Do the pages load fast enough?							
*Answers*	*1*	*1*	*1*	*3*	*25*	*31*	*4.61*	*92.2%*
9	Did you have any other technical difficulties?							
Scale	Yes		No					
*Answers*	*4*		*6*			*10*		*60%*
10	Is the content of the pictures visible and the resolution good enough?							
*Answers*	*1*	*3*	*1*	*9*	*14*	*28*	*4.41*	*88.2%*

## Results

### The Mummy Explorer

**Figure 1.**
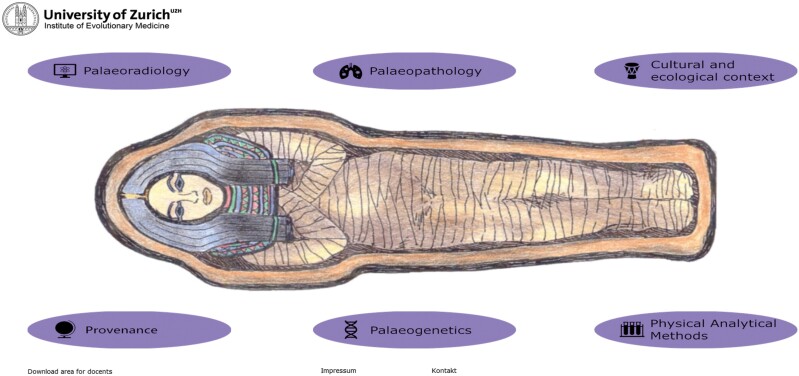
Front page of the *Mummy Explorer*

The tool starts with a front page (Fig. 1). It contains information about six subtopics: paleoradiology, palaeopathology, cultural and ethnological context, provenance, paleogenetics, and physiological analytical methods. These topics are related to several lectures and research projects at the Institute for Evolutionary Medicine (IEM) at the University of Zurich (UZH). All the texts and information used in the *Mummy Explorer* were provided by lecturers of the courses that the tool is supposed to substitute or support. Each of the topics has its own subpage and provides references to the information given. For a better understanding, the contents are presented using the example of investigations on a mummy. One can see, for example, the analysis of vascular diseases using X-rays under the subtopic of paleoradiology. This type of analysis is further linked to the subtopic of palaeopathology to show the interconnection. Similarly, other topics, such as protein analysis and ancient DNA analysis of dental calculus, are both connected to the analysis of past diets, which is also part of the subtopic of the analysis of stable isotopes. Additionally, more cultural aspects, such as the provenance and the cultural context of the mummy, are discussed to reconstruct the life of the studied individual as much as possible and to show the students the large variety of potential research questions.

**Figure 2.**
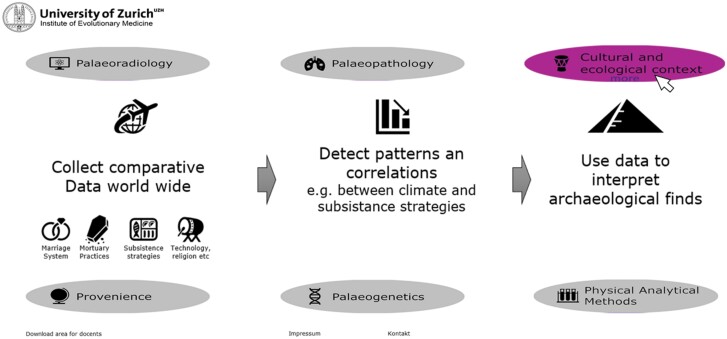
Interactive mouse-hover events on the front page of the *Mummy Explorer*

When hovering over one of the subtopic buttons on the front page, a schematic summary of the content appears (Fig. 2). Once the button is clicked, a subpage opens with more detailed content about the subtopic. Each subpage contains a text with an overview of the research field provided by the lecturers on this topic and pictures or schemes to further clarify the content. The subtopics are linked to each other with hyperlinks to clarify the connections to the students. For each subtopic, informative illustrations and summary texts are provided. All figures are animated, and hovering over them gives additional information in the form of legends. The illustrations are also used for interlinking the connected subtopics. The information about which subtopic might be linked appears when hovering over the figure, and clicking will automatically forward the reader to the referenced section. Furthermore, for each section, external hyperlinks and citations of papers and books are provided for further reading so that the students can independently deepen their knowledge.

In this way, the students are provided with an opportunity to explore the content of the *Mummy Explorer* by themselves. The link to the tool is provided to the students at the beginning of each lecture series and accompanies the lectures’ contents. This means that the tool’s content is not entirely new to them, but they should use it to deepen their knowledge and prepare for their exams.

The online tool is available at https://www.iem.uzh.ch/static/Mummy_explorer/.

### Template for lecturers

The template provided for lecturers contains the front page and pages for the subtopics. The front page is already animated. The template is filled with placeholders for pictures and text blocks. All the lecturers have to adapt the tool for their own lectures by swapping out the pictures, replacing the text and links, and finding a solution for hosting the tool (see the methods section below for suggested solutions).

The downloadable folder with the template also contains a manual with instructions (see Supplementary Information material). It starts with the free software installation and then guides the users through every step necessary to adjust the template to their own needs, such as replacing space holders for pictures and entering their own text. There is a description of how the lecturers can interlink the subpages according to their requirements. The template and the manual were also tested with a group of students and interested lecturers (Table 2). The survey allowed us to improve the manual for users who were not familiar with the software (see the following section).

### Survey

In 2021, 233 students and in 2022, 191 students from five different courses (one course from the Medical Faculty and four courses from the Natural Sciences Faculty of the University of Zurich) were invited to test the online tool. In 2021, 67 (28.8%) students participated in the survey and answered the questions (Table 1). The majority of the answers were positive regarding the usefulness of the tool. Specifically, the tool provided a good overview of possible research areas (98.5% positive), as well as the kind of data that can be collected and for what purpose (98.5% positive). The links between the subtopics were understandable (75% positive), and the number of illustrations and schemes was sufficient (78.5% positive). Overall, the tool was described as understandable, and students saw it as a helpful addition to the lectures. Technical issues were less common. A total of 7.8% could not open the tool immediately, while in 20.3% of the cases, the website did not load quickly, and in 3.1% of the cases, the pictures were not visible.

In the survey, there was also space for additional comments. This section of the survey provided valuable comments for improvement, for example, to create a hyperlink for the UZH logo, which then directs to the UZH web page and other minor technical and content-related suggestions, such as a higher resolution front-page figure, or more hyperlinks in the various subpages.

**Figure 3.**
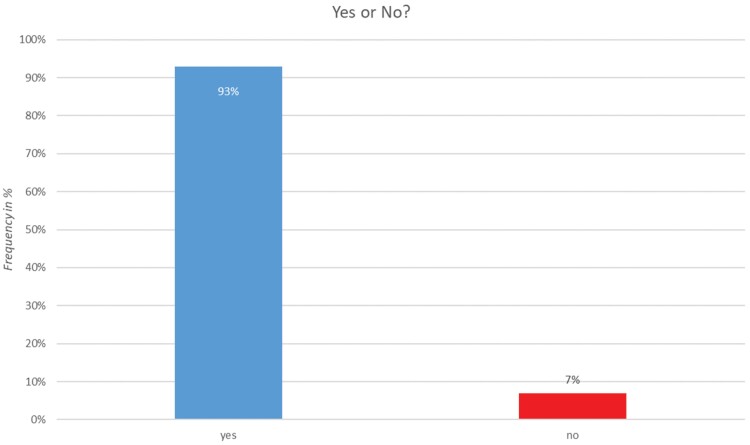
Answers of the lecturers to the question: Do you think providing a template is useful, and can you think of other lectures where such a tool could be useful?

Lecturers testing the template for setting up their own tool answered questions in a separate survey. A total of 15 lecturers gave feedback (Table 3). Over 90% of the lecturers who tested the template answered that they could imagine using it for their own teaching and found the idea useful (Fig. 3). Technical issues were less common. In 6.7% of cases, it was not possible to download the program; in 15.4%, there were problems with downloading the template or opening it in the Google Web Designer. A total of 61.5% found the instructions to be unclear. Comments about the instructions were especially useful and helped to improve the description of the process so that in a second survey round, everyone could use the template, for example, by finding the correct button to change the placeholder text.

**Table 3.** Questions and answers to the 2021 survey for the lecturers. The total number of participants in the survey was 15 lecturers who used the template to create a similar online tool.

Question Nr	Question	Number of answers	Nr Yes	% Yes
1	Could you download the software?	15	14	93.3%
2	Could you download the template and open it in the Google Web Designer?	13	11	84.6%
3	Are the steps in the manual sufficiently explained?	13	5	38.5%
4	Which steps need more details?	12	N/A	
5	What problems did you experience when setting up your own version?	12	N/A	
6	Do you think providing a template is useful and can you think of other lectures where such a tool could be useful?	13	12	92.3%

In 2022, we repeated the survey with additional students and a different answer scheme with a Likert scale of 1–5 instead of binary responses (Table 2, Supplementary Information Note IV). Of the 191 students invited to participate in the second survey, 34 participated (18%). As in the previous year, the content and technical questions were answered positively (predominantly 4 or 5 points), including the new question, ‘Do you think the *Mummy Explorer* can support your learning experience?’. The majority of students [18] answered this question in the highest categories 4 and 5, while five students answered it in the lowest categories 2 and 1.

## Discussion

We developed a novel, interdisciplinary online teaching tool, the *Mummy Explorer,* to better interlink and explain the contents of complex and diverse lecture series such as evolutionary medicine. With two surveys conducted among students and lecturers at the UZH, we explored the performance of the content and technical aspects of the tool. The first survey was answered by more students than the second survey (67 compared to 34), and the result was slightly more positive in the first one. This discrepancy could be explained by the use of a Likert scale instead of binary answer categories in the second survey. As the surveys were fully anonymous we could not analyse the heterogeneity of answers further, for instance to analyse if the answers were clustered by course or by teacher. Furthermore, the rather low response rate does not allow to exclude a bias towards positive answers in both survey years.

All participating students could use the tool, and technical problems were solved quickly so that the full benefit of the tool could be assessed. Overall, the content was rated as understandable, and the illustrations and animations were rated as interesting and helpful. The surveys showed that the students were positive about the tool and felt it could significantly contribute to their learning success. For instance, they confirmed that the tool provided materials and external links to better understand the lectures and an overview of how the different subtopics were interlinked. Likewise, the lecturers found the tool interesting, and they could imagine using it in their own teaching.

The technical challenges were more demanding. Due to the received feedback, technical and graphical details were adjusted, for example, changing the picture on the front page or adding figure legends. The second survey showed that the improvements increased the user-friendliness, such as the loading speed of the tool. The surveys furthermore showed that, especially for the lecturers, the instructions had to be adjusted to increase user-friendliness. This point is of major relevance.

Since open-access digital teaching should and must be further developed in the future, one main goal of the project was to design the tool to be adapted for other subject areas. Thus, substituting a skeleton, a living human, a patient, a child, an ape, or even a theoretical concept instead of the mummy could make the central topic being considered more apparent to users. We, therefore, provide the template using free software and with the possibility of hosting the resulting tool via a free Google account. This makes the tool usable for a wide range of lecturers and tutors even if they have no opportunity to publish their tool with university services. The free piece of software with an intuitive GUI enables lecturers to create their own tools quickly and with little effort. This approach aligns with the UNESCO Open Education Resources initiative [17] and reflects the increasing demand for open online education [18].

Especially in complex fields such as evolutionary medicine, applying a teaching tool facilitating interconnection and a deeper understanding of content might be very helpful. Problems with existing encyclopaedic platforms such as Wikipedia are that they are not necessarily curated by experts, and hence, the information provided might not be accurate. Furthermore, large platforms usually contain a lot of information, of which only parts are relevant to the students of specific lectures. Our tool provides students with the exact information to deepen their knowledge or prepare for exams. Including evolutionary concepts in medical education and aligning them with learning objectives and competencies is expected to enhance critical thinking among physicians [8]. Some positive examples exist. For instance, evolutionary medicine is taught at the Medical Faculties of the Universities of Bern and Zurich in Switzerland. It has also been successfully introduced in the curriculum of medical schools in Pakistan [19]. A survey among nutrition and dietetics professionals showed a high interest in incorporating evolutionary medicine into this field [20].

Evolutionary biology can be introduced into the medical curriculum according to Bloom’s revised taxonomy [8]. According to this taxonomy, the six levels of learning include the skills that the *Mummy Explorer* also promotes: level 1: remembering; level 2: understanding; level 3: applying; level 4: analysing; level 5: evaluating; and level 6: creating. While levels 1–2 can be achieved by studying the contents of the online tool, levels 3–6 can be achieved by reflecting on the contents and the interconnections between them, by asking questions and giving feedback to the teacher and other students, and by creating one’s own ideas about applications. In the example of the mummy excavation, this could be a protocol for a lab experiment, a plan for a field expedition, or a differential diagnosis for palaeopathological findings. These latter tasks can also be developed as the students work through collaborative tasks. Furthermore, the contents of the online tool can be used as a repetition tool or a learning control for lectures.

To ensure the design of digital teaching materials in a way that fits the needs of the students as much as possible, it is important to integrally involve them in the development process. The *Mummy Explorer* was assessed by students who were attending the lectures at which the tool was targeted. It is, therefore, not surprising that most of the students who tested the tool found it helpful. Future applications of the tool with students of other courses or universities can give feedback on the tool to constantly improve its format and function.

Furthermore, an active learning format should be sought whenever possible. Active learning promotes skills such as the practical application of concepts, analysis of data, and the creation of novel knowledge synthesis, according to the higher levels of Bloom’s taxonomy [21]. A review showed that active learning is very efficient in teaching evolutionary content [1]. As a practical example, students can develop new versions of the *Mummy Explorer* to create new content as part of an active and constructivist learning approach. Such didactical techniques have been shown to be especially efficient [22]. Creating a new version of the explorer can be done as an exercise with the students, thus allowing them not only to choose the appropriate content for the learning platform but also to meaningfully interlink them. In the cycle of self-regulated learning, this task demands proper planning (preparation) of the contents, a well-thought implementation by creating the tool (performance), and a thorough appraisal of the result (appraisal) [3].

One possible difficulty that needs to be addressed in individual virtual learning is the reduced interaction with peers and teachers. Although the *Mummy Explorer* can also be used to accompany the class, its primary use is probably an asynchronous, individual application by students. A feedback form is included in the tool, but direct interaction, especially with peers, is not possible. This difficulty is common to many virtual teaching tools. It can best be addressed by combining virtual teaching tools such as the *Mummy Explorer* with live in-class discussions about the learned content, integrated peer group works, forums, and other similar didactical techniques.

## CONCLUSIONS AND IMPLICATIONS

The coronavirus pandemic and the resulting lockdowns, during which face-to-face teaching at universities had to be suspended, once again highlighted the importance of expanding digital education. However, in digital teaching, where there is no direct contact with the student, it is difficult to address the individual problems of the students. The teaching format must therefore be chosen in a way that is as intuitive as possible for the students. Tools such as the *Mummy Explorer* might offer flexible applications to combine on-site and online didactical elements in a blended learning fashion to increase active and self-regulated learning experiences. Importantly, the *Mummy Explorer* further adds the capacity to interlink complex contents and is adaptive to many applications and scientific fields.

The next steps within the IEM are to translate the *Mummy Explorer* into German and to create a new version of the tool with a living human in the centre, with related evolutionary medicine topics surrounding it. This new version will be a student project developed within a practical course at the IEM. All versions will be available on the institute’s website for free download (https://www.iem.uzh.ch/static/Mummy_explorer/). Furthermore, we will continue to survey the performance of the tool with surveys among students and lecturers.

## Supplementary Material

eoad009_suppl_Supplementary_Material
