# Supplementary material for: The Mummy Explorer—a self-regulated open-access online teaching tool
Source: Evol Med Public Health. 2023 Apr 27;11(1):129–38. doi: 10.1093/emph/eoad009 (PMC10224693; doi:10.1093/emph/eoad009)
Supplement: eoad009_suppl_Supplementary_Material [file eoad009_suppl_supplementary_material.pdf]

Inhalt

SI Note I Manual Mummy Explorer 2

SI Note II Results of the first Student Questionnaire 8

SI Note III Results of the Lecturers Questionnaire 20

SI Note IV Results of the Second Student Questionnaire 23

SI Note I Manual Mummy Explorer

1. Download Google Web Designer from here: https://webdesigner.withgoogle.com/

2. Download the Mummy Explorer Template from the website: Then extract the zip folder and save it in the folder where you deposited the Google Web Designer as for example: “C:\Users\afurtw\Google Web Designer\templates” *Note: This is not the folder under programs, the Google Web Designer folder is generated automatically and contains already two subfolders templates and components, it could be for example under user or in documents, it does not work to create the folder yourself*
In the folder templates should be one folder for each template:

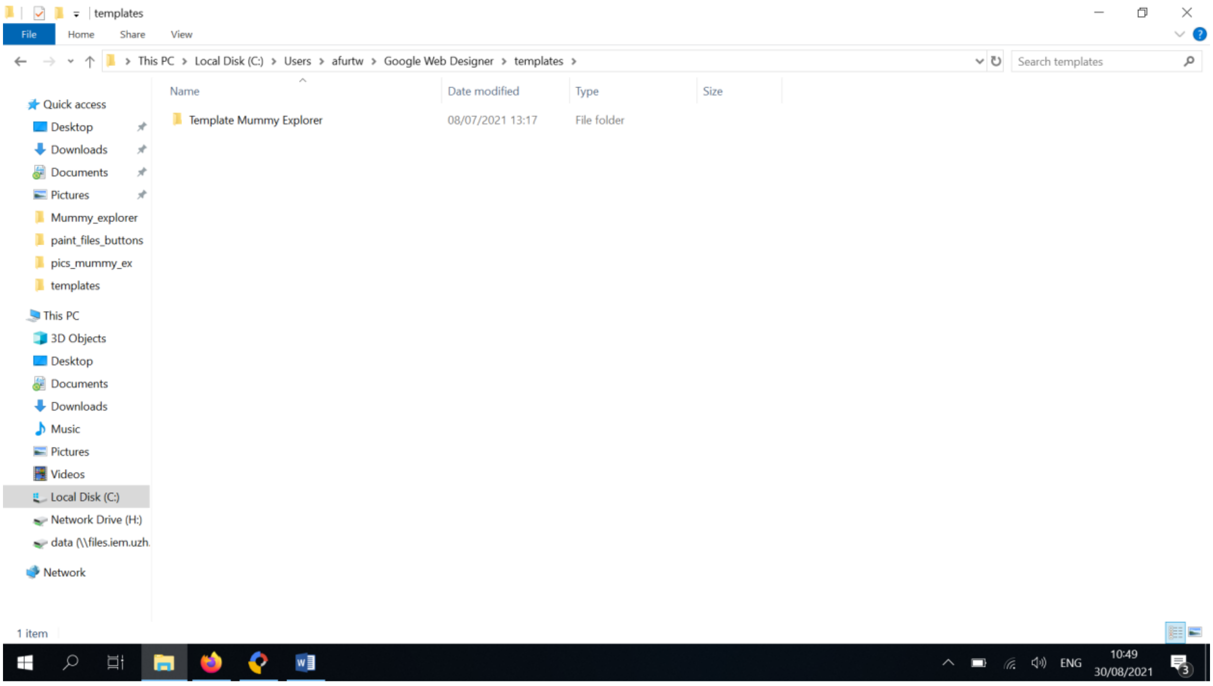

In the template folder should be the content of the .zip folder

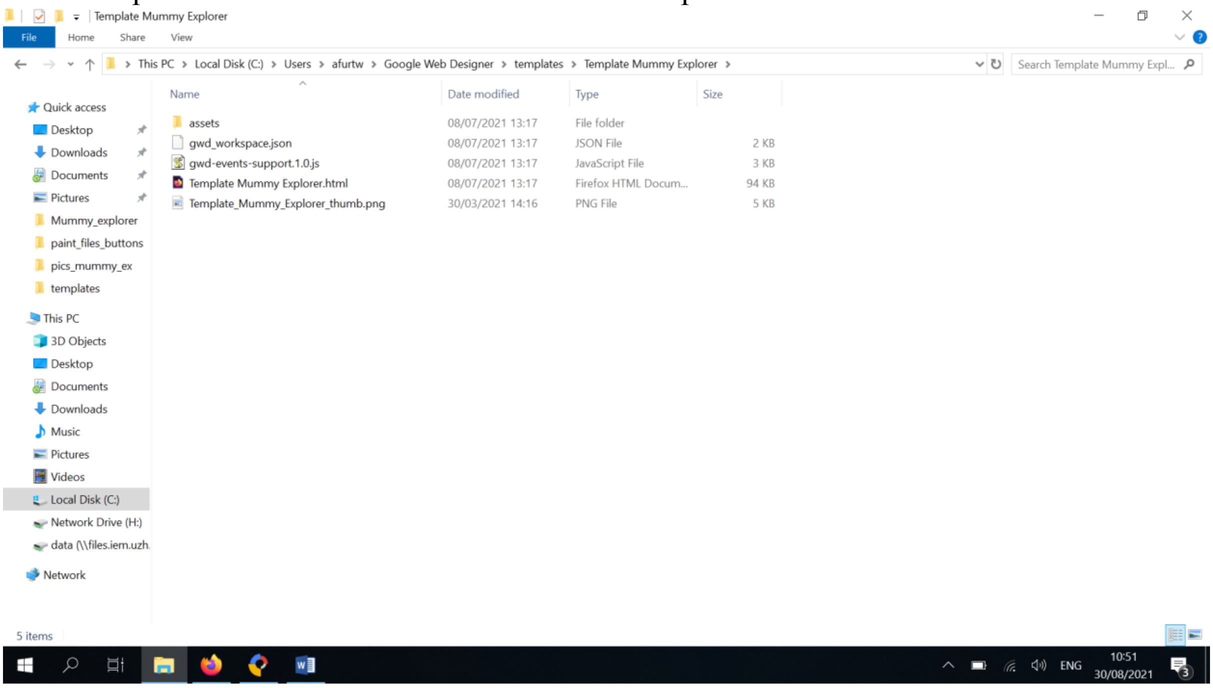

3. Open the Google Web Designer, chose the Option *Use Template*

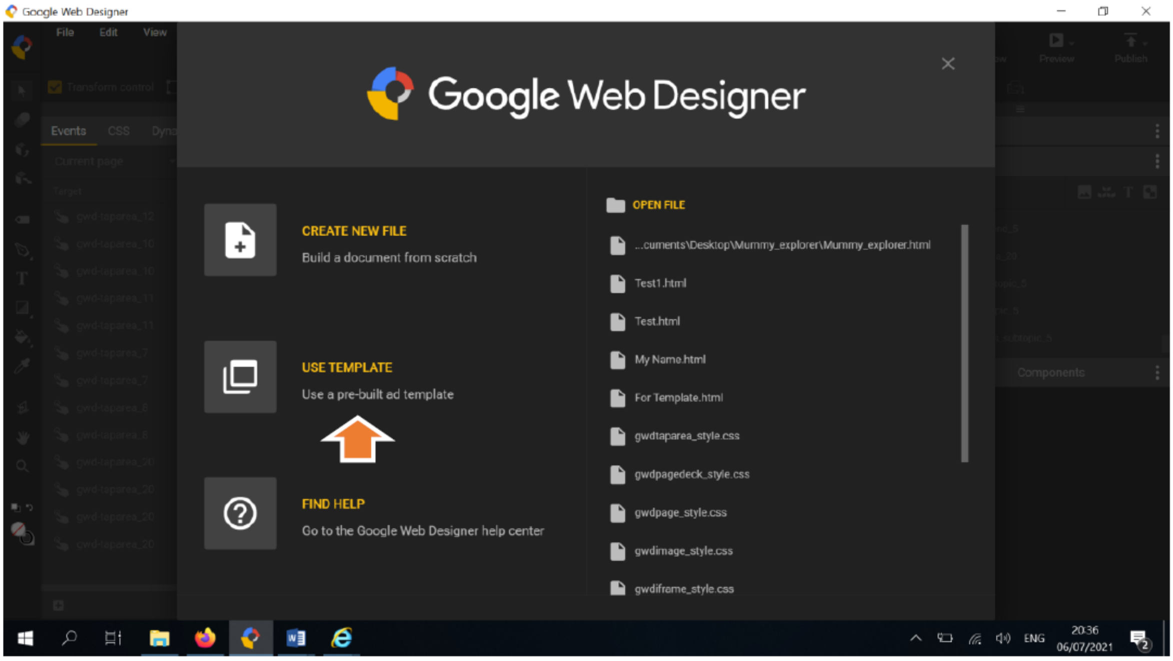

4. Select My Templates and then select the Mummy Explorer Template

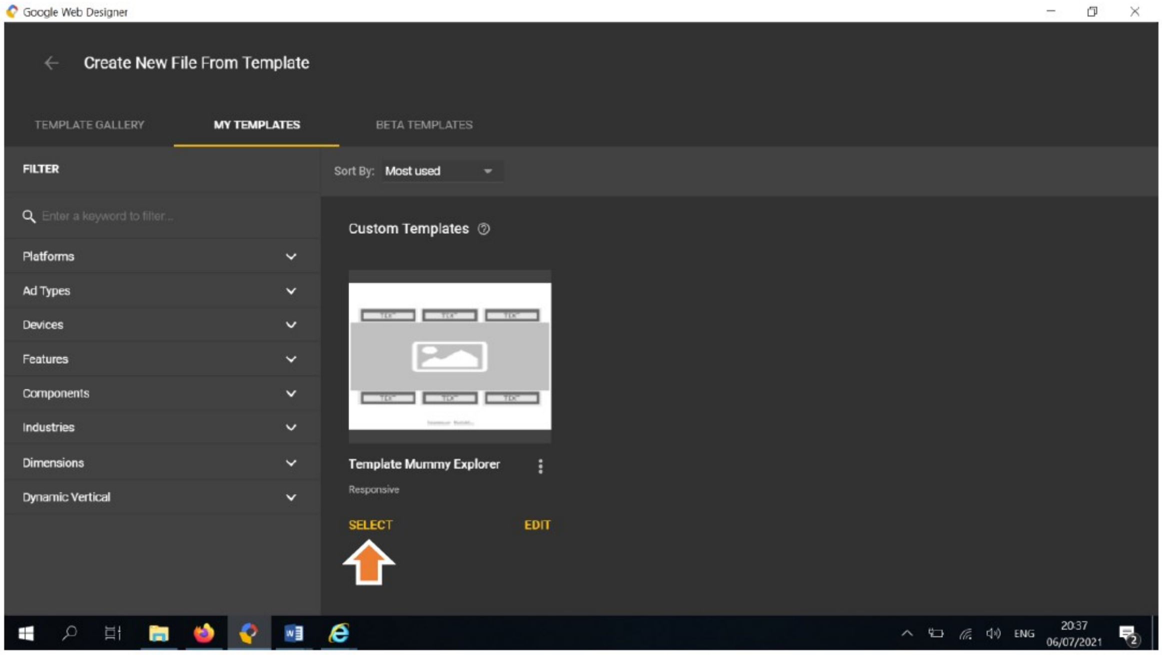

5. Give your file a meaningful name and click create

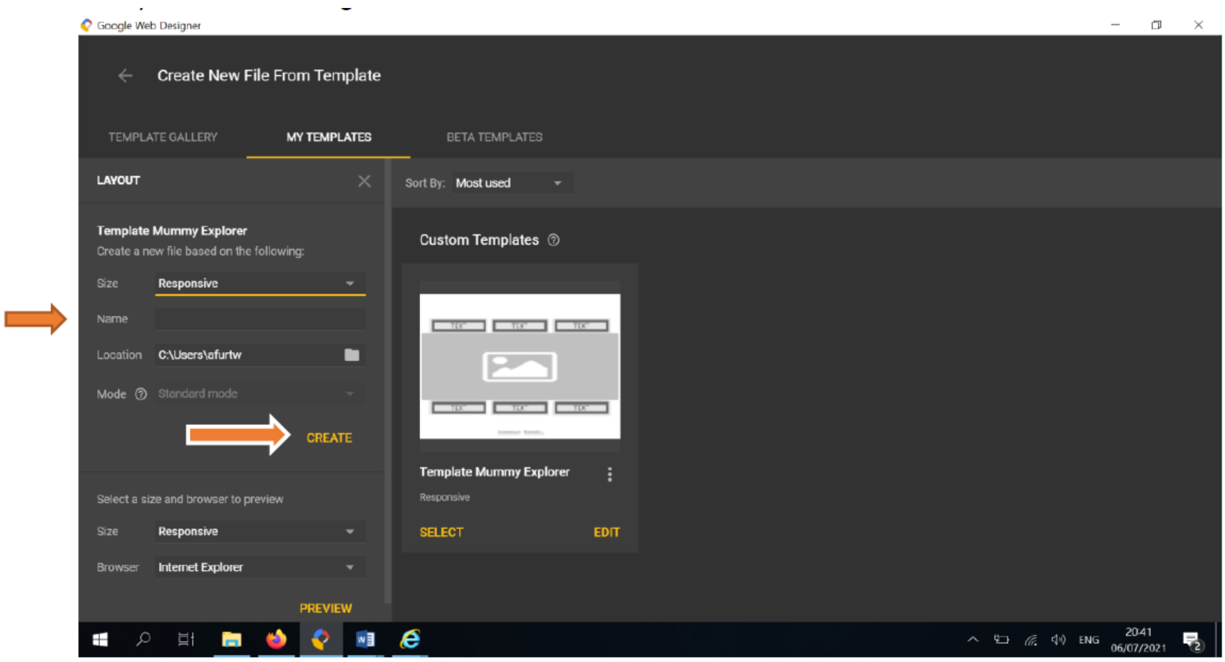

6. You can find a detailed manual for the Google Web Designer online: https://support.google.com/webdesigner

7. How to prepare the content of your webpage: -The text on the subtopic buttons on the title page can not be changed. You need to prepare a graphic for each button with an aspect ratio of 3:1 Aspect ratio of pictures: -Title picture 10:5 -Subtopic overview of title page 10:5 -Picture Subtopic 4:5

8. Where to find everything: Different subpages can be found in the page deck and the different components can be found in the outliner

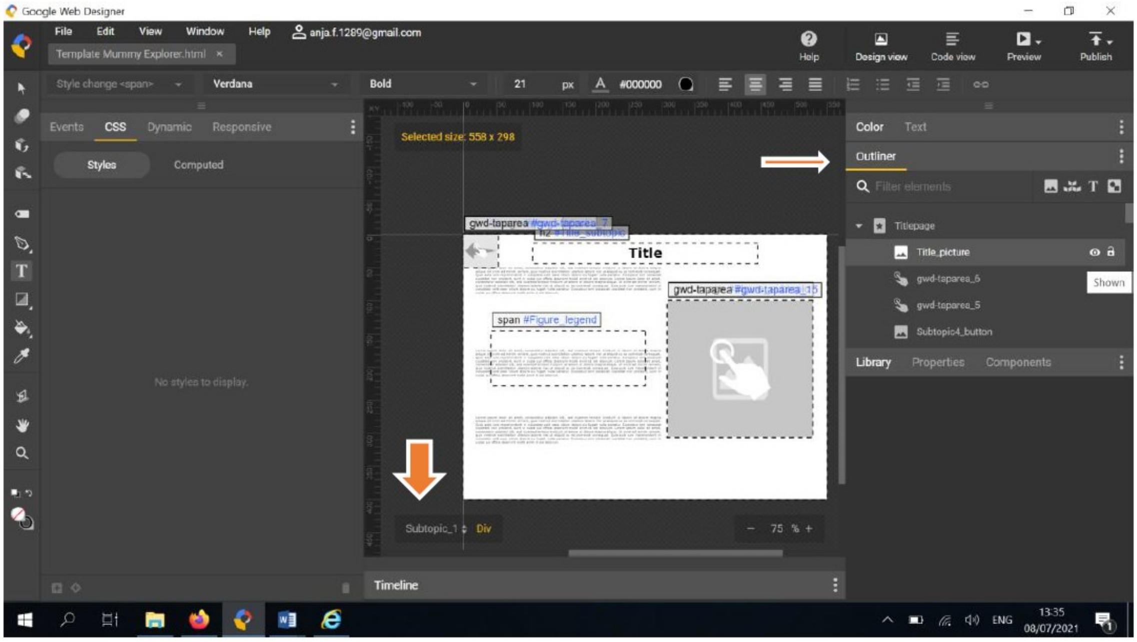

9. Load all images you want to use into the library

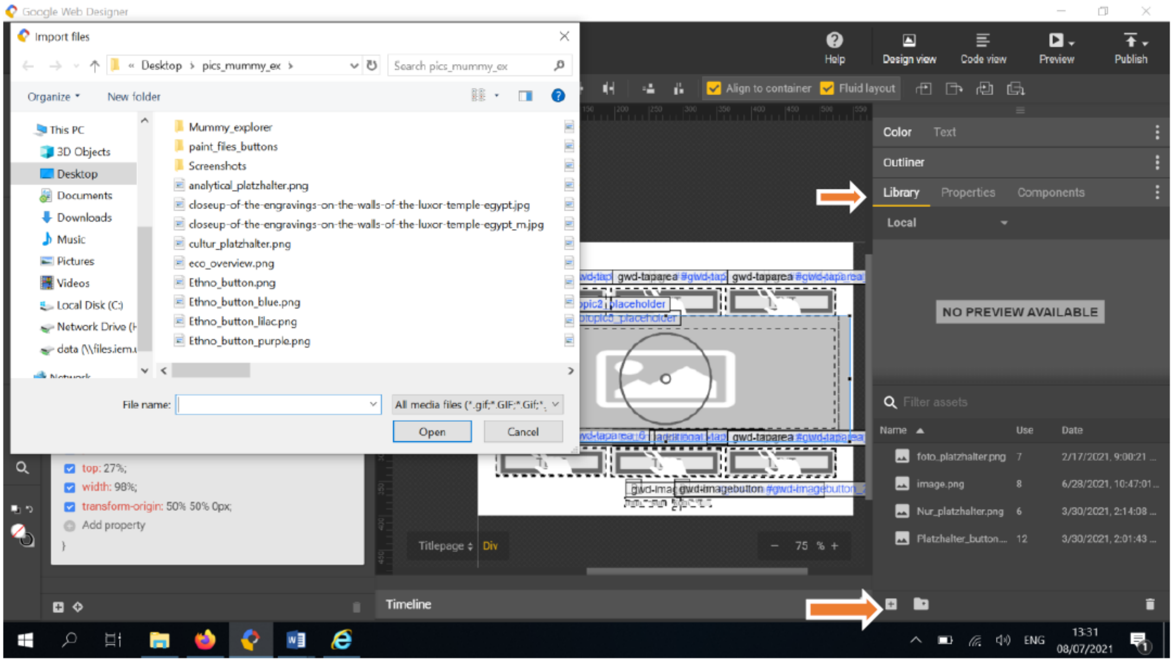

10. Exchange pictures on the titlepage right click on the picture and select swap image and select the image from the drop down menu

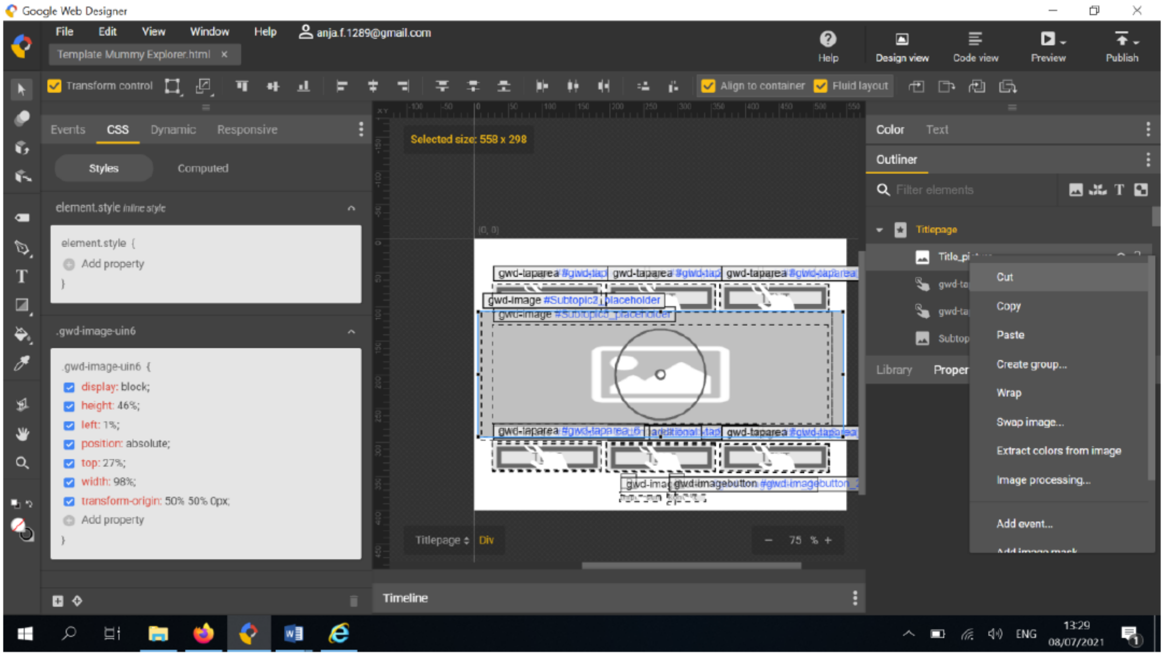

11. Change text: Click on the text tool and mark the text you want to replace, start writing or paste copied text, once you are done click back to the selection tool to continue working on other objects

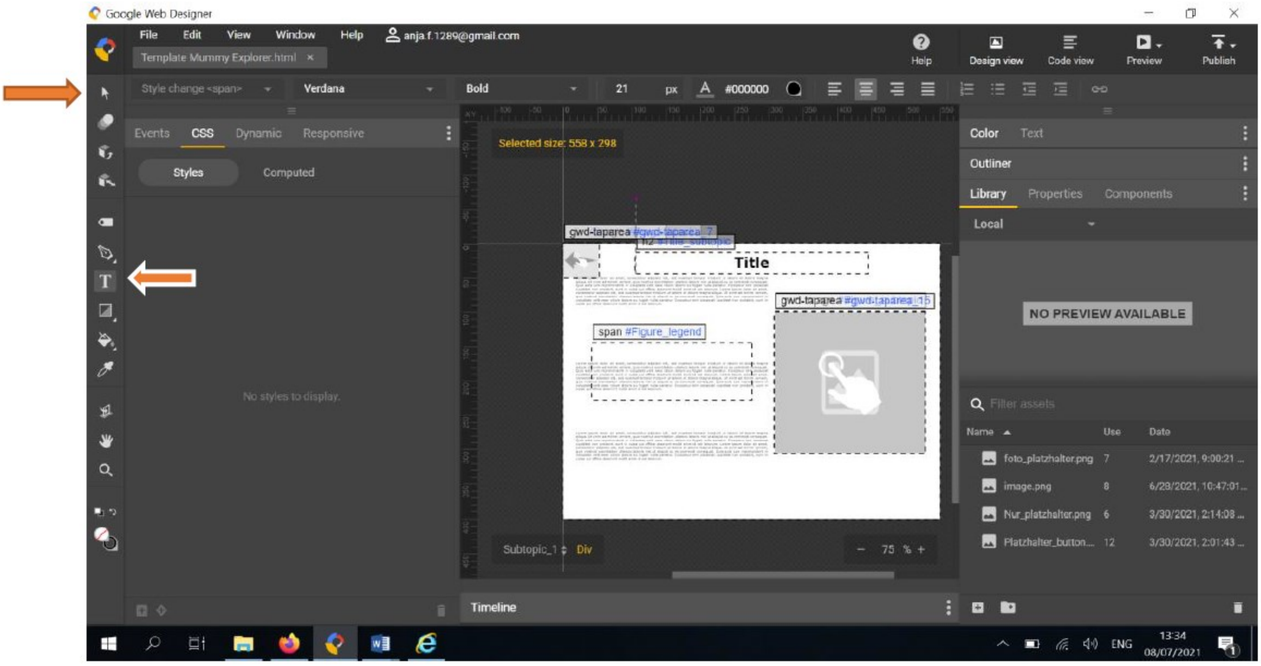

12. Change the text of figure legend: The figure legend of each page is invisible and only displayed when hovering over the picture. This only works in the final published webpage or in the preview. To change the text the figure legend has to be made visible, then the text can be changed in the same way as any other text. Do not forget to make the figure legend invisible after changing the text. Otherwise the texts will overlap in the final webpage.
To make the legend visible click on it, navigate to properties: visibility and change from hidden to visible, replace text with your own, change back to hidden after changing the text

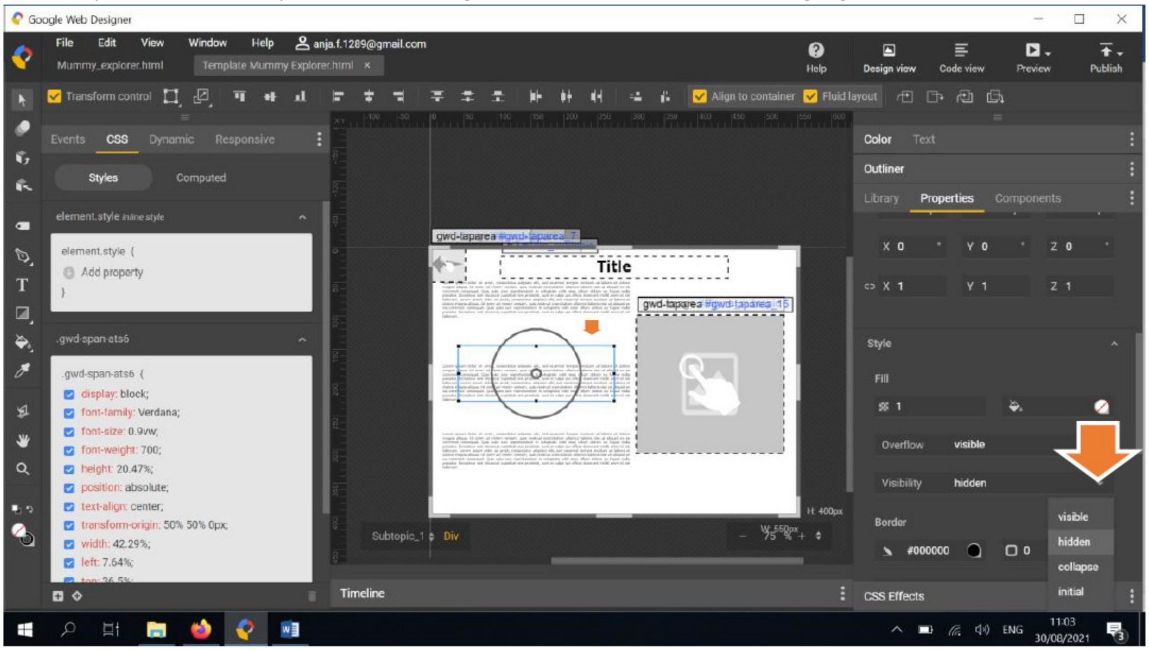

13. Always make sure that the size of every object is in percentage:

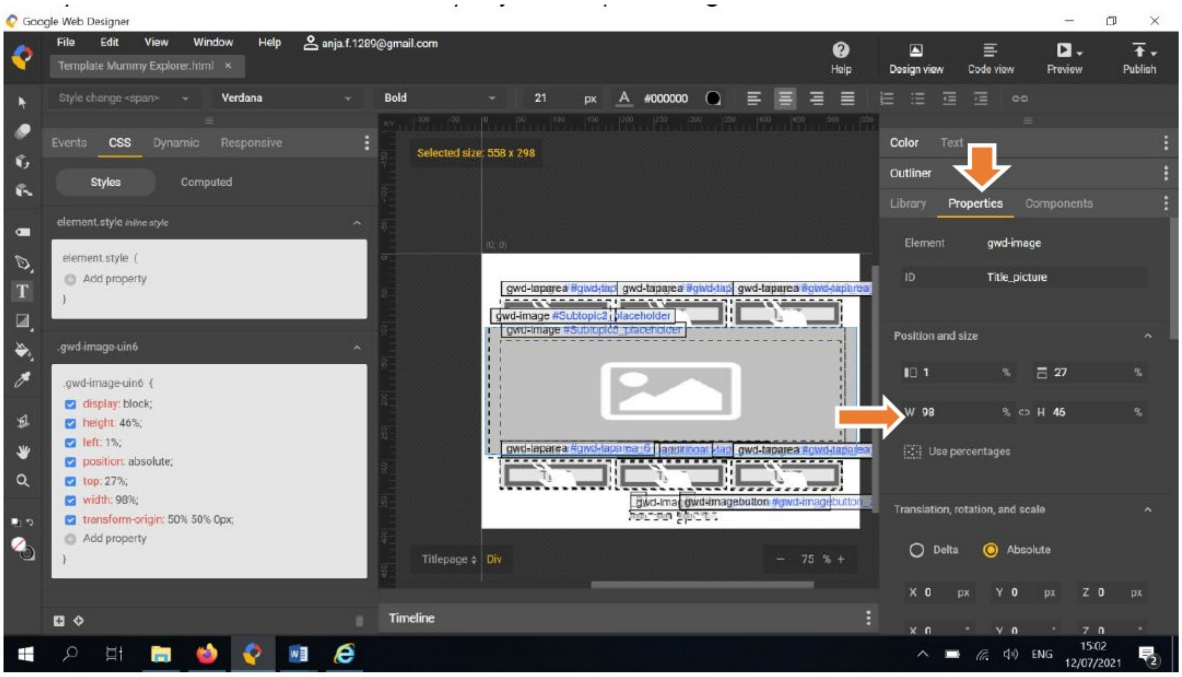
Google Web Designer
File
Edit
View
Window
Help
anja.f.1289@gmail.com
Template Mummy Explorer.html
Help
Design view
Code view
Preview
Publish
Verdana
Bold
21
px
#000000
Events
CSS
Dynamic
Responsive
Styles
Computed
element.style inline style
element.style {
Add property
}
.gwd-image-uin6
.gwd-image-uin6 {
display: block;
height: 46%;
left: 1%;
position: absolute;
top: 27%;
width: 98%;
transform-origin: 50% 50% 0px;
Add property
}
Selected size: 558 x 298
Titlepage
Div
75 %
Timeline
Color
Text
Outliner
Library
Properties
Components
Element
gwd-image
ID
Title_picture
Position and size
1
27
W 98
H 46
Use percentages
Translation, rotation, and scale
Delta
Absolute
ENG
15:02
12/07/2021

SI Note II Results of the first Student Questionnaire

The survey for the students was performed with lamapoll. It was split in two rounds for technical reasons. The free version of lamapoll only allows to collect the results of 50 participants.

Results 1st round:

Participation overview

	Quantity	Quote
Visitors	76	-
Participations	50	65.79%
Returns	47	94.00%
Dropout	3	6.00%

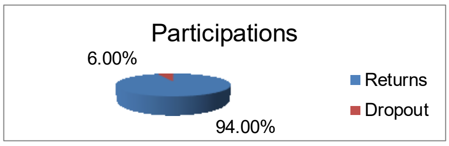

Participant	launched	ends	aborted
Anonymous	50	47	3
Access key	0	0	0
Address book	0	0	0
**Total**	**50**	**47**	**3**

Question 1 - Yes or no? Does the tool provide an overview of possible analysis for the study of mummies?

Booth: May 30, 2022, 11:25 AM, Survey "Mummy-Explorer"

Number of participants evaluated: 50 (all participants)

Created with LamaPoll | https://www.lamapoll.de

Status data

from 50 Participant	Quantity	Percent
Question seen	50	100.00%
Question answered	50	100.00%
Question not answered	0	0.00%

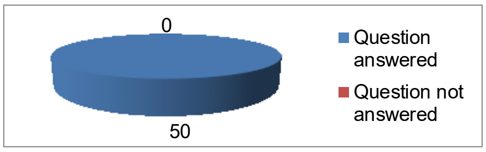

Results

Options	Quantity	Frequency
Yes	49	98.00%
No	1	2.00%
**Total**	50 Answer	50 Participant

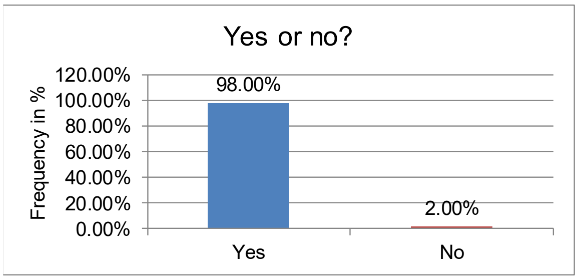

Question 2 - Yes or no? Did you get an idea of what data can be collected and for what purpose?

Booth: May 30, 2022, 11:25 AM, Survey "Mummy-Explorer"

Number of participants evaluated: 50 (all participants)

Created with LamaPoll | https://www.lamapoll.de

Status data

from 50 Participant	Quantity	Percent
Question seen	50	100.00%
Question answered	49	98.00%
Question not answered	1	2.00%

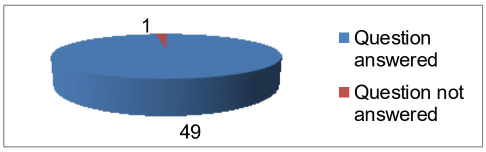

Results

Options	Quantity	Frequency
Yes	48	97.96%
No	1	2.04%
**Total**	49 Answer	49 Participant

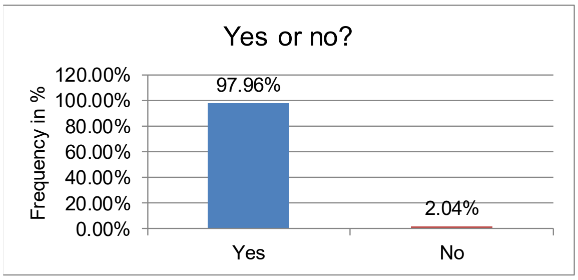

Question 3 - Yes or no? Does the tool contain enough pictures and schemes?

Booth: May 30, 2022, 11:25 AM, Survey "Mummy-Explorer"

Number of participants evaluated: 50 (all participants)

Created with LamaPoll | https://www.lamapoll.de

Status data

from 50 Participant	Quantity	Percent
Question seen	49	98.00%
Question answered	48	96.00%
Question not answered	2	4.00%

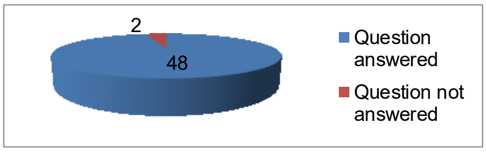

Results

Options	Quantity	Frequency
Yes	38	79.17%
No	10	20.83%
**Total**	48 Answer	48 Participant

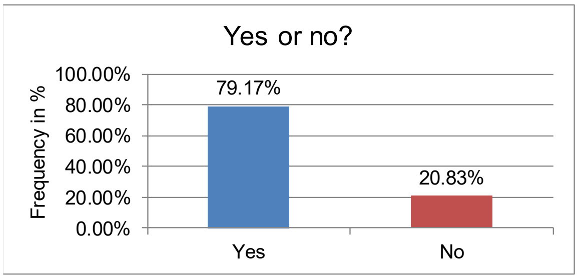

Note: Additional pictures were added after this survey.

Question 4 - Yes or no? Did you get an idea of how the different research areas are linked?

Booth: May 30, 2022, 11:25 AM, Survey "Mummy-Explorer"

Number of participants evaluated: 50 (all participants)

Created with LamaPoll | https://www.lamapoll.de

Status data

from 50 Participant	Quantity	Percent
Question seen	48	96.00%
Question answered	47	94.00%
Question not answered	3	6.00%

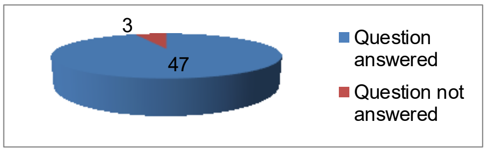

Results

Options	Quantity	Frequency
Yes	34	72.34%
No	13	27.66%
**Total**	47 Answer	47 Participant

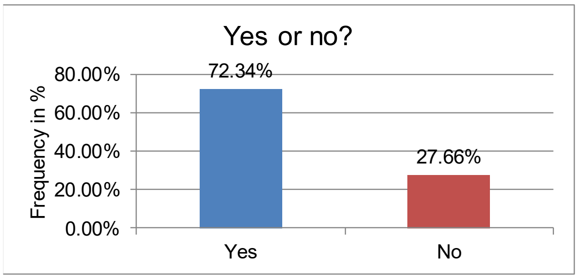

Note: Hyperlinks between the subtopics were created after this survey.

Question 5 - Yes or no? Are enough references for further reading provided?

Booth: May 30, 2022, 11:25 AM, Survey "Mummy-Explorer"

Number of participants evaluated: 50 (all participants)

Created with LamaPoll | https://www.lamapoll.de

Status data

from 50 Participant	Quantity	Percent
Question seen	47	94.00%
Question answered	46	92.00%
Question not answered	4	8.00%

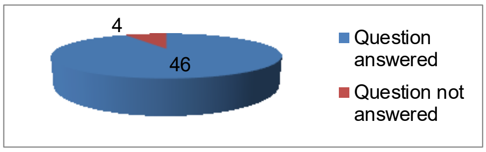

Results

Options	Quantity	Frequency
Yes	41	89.13%
No	5	10.87%
**Total**	46 Answer	46 Participant

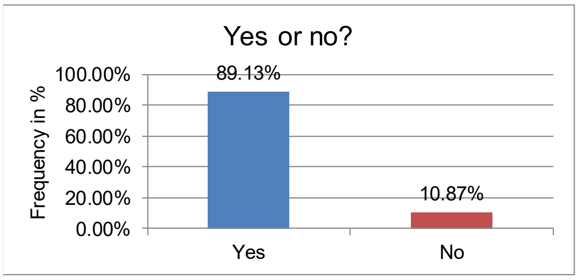

Question 6 - Please fill in the answer. Is there something you did not understand? Why?

Booth: May 30, 2022, 11:25 AM, Survey "Mummy-Explorer"

Number of participants evaluated: 50 (all participants)

Created with LamaPoll | https://www.lamapoll.de

Status data

from 50 Participant	Quantity	Percent
Question seen	47	94.00%
Question answered	13	26.00%
Question not answered	37	74.00%

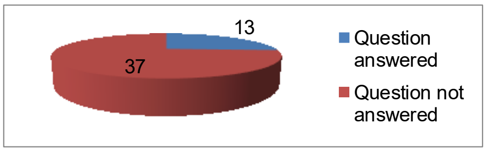

For data protection reasons individual answers are not shown but the majority of the students that answered the question confirmed that they understood the content. Which also were addressed in the following questions.

Question 7 - Yes or no? Could you open the tool?

Booth: May 30, 2022, 11:25 AM, Survey "Mummy-Explorer"

Number of participants evaluated: 50 (all participants)

Created with LamaPoll | https://www.lamapoll.de

Status data

from 50 Participant	Quantity	Percent
Question seen	47	94.00%
Question answered	47	94.00%
Question not answered	3	6.00%

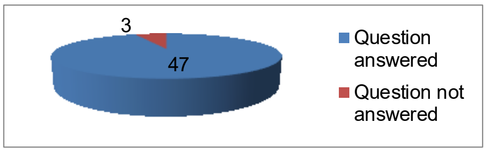

Results

Options	Quantity	Frequency
Yes	42	89.36%
No	5	10.64%
**Total**	47 Answer	47 Participant

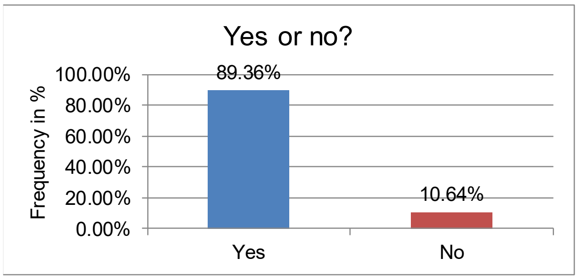

Note: Some mobile devices do not support the format.

Question 8 - Yes or no? Do the pages load fast enough?

Booth: May 30, 2022, 11:25 AM, Survey "Mummy-Explorer"

Number of participants evaluated: 50 (all participants)

Created with LamaPoll | https://www.lamapoll.de

Status data

from 50 Participant	Quantity	Percent
Question seen	47	94.00%
Question answered	47	94.00%
Question not answered	3	6.00%

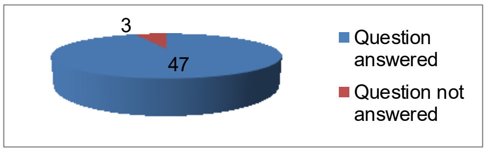

Results

Options	Quantity	Frequency
Yes	38	80.85%
No	9	19.15%
**Total**	47 Answer	47 Participant

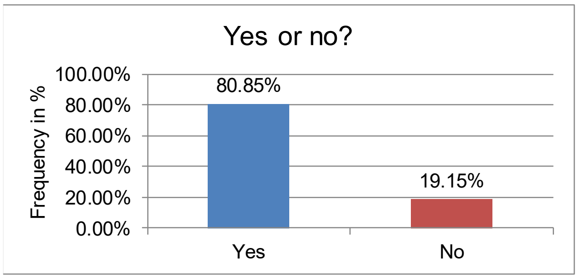

Note: Tests were performed while hosting on a google drive. The version on the IEM server loads faster.

Question 9 - Please fill in the answer. Did you have any other technical difficulties?

Booth: May 30, 2022, 11:25 AM, Survey "Mummy-Explorer"

Number of participants evaluated: 50 (all participants)

Created with LamaPoll | https://www.lamapoll.de

Status data

from 50 Participant	Quantity	Percent
Question seen	47	94.00%
Question answered	22	44.00%
Question not answered	28	56.00%

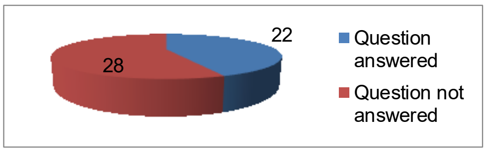

For data protection reasons individual answers are not shown. Mentioned were the speed in which the pages were loading and a few formatting problems with overlapping texts which were addressed after the text.

Question 10 - Yes or no? Is the content of the pictures visible and the resolution good enough?

Booth: May 30, 2022, 11:25 AM, Survey "Mummy-Explorer"

Number of participants evaluated: 50 (all participants)

Created with LamaPoll | https://www.lamapoll.de

Status data

from 50 Participant	Quantity	Percent
Question seen	47	94.00%
Question answered	47	94.00%
Question not answered	3	6.00%

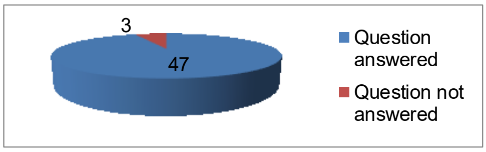

Results

Options	Quantity	Frequency
Yes	45	95.74%
No	2	4.26%
**Total**	47 Answer	47 Participant

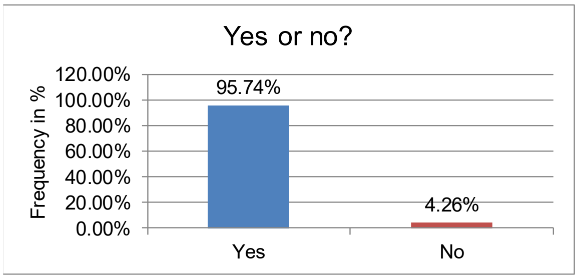

Question 11 - Please fill in the answer. Space for more comments. What did you like? What should be improved?

Booth: May 30, 2022, 11:25 AM, Survey "Mummy-Explorer"

Number of participants evaluated: 50 (all participants)

Created with LamaPoll | https://www.lamapoll.de

Status data

from 50 Participant	Quantity	Percent
Question seen	47	94.00%
Question answered	20	40.00%
Question not answered	30	60.00%

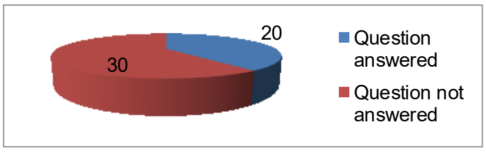

For data protection reasons individual answers are not shown. Points that were raised: Link to the IEM webpage for the logo, More pictures for some subtopics, More connections between the subtopics, Zoom in for the picture was requested

Results 2nd round:

Participation overview

	Quantity	Quote
Visitors	40	-
Participations	18	45.00%
Returns	18	100.00%
Dropout	0	0.00%

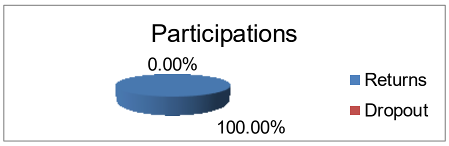

Participant	launchec	ends	aborted
Anonymous	18	18	0
Access key	0	0	0
Address book	0	0	0
**Total**	**18**	**18**	**0**

Question 1 - Yes or no? Does the tool provide an overview of possible analysis for the study of mummies?

Booth: May 30, 2022, 11:26 AM, Survey "Mummy-Explorer-2"

Number of participants evaluated: 18 (all participants)

Created with LamaPoll | https://www.lamapoll.de

Status data

from 18 Participant	Quantity	Percent
Question seen	18	100.00%
Question answered	18	100.00%
Question not answered	0	0.00%

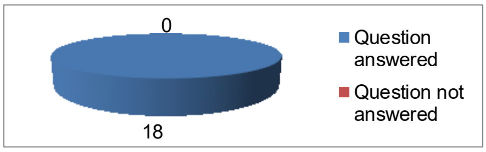

Results

Options	Quantity	Frequency
Yes	18	100.00%
No	0	0.00%
**Total**	18 Answer	18 Participant

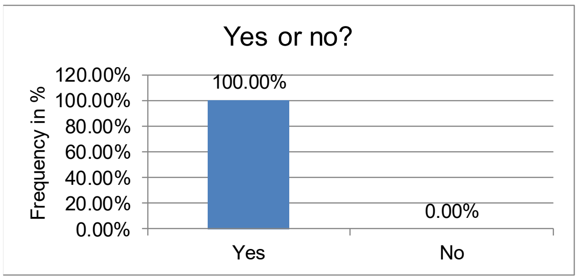

Question 2 - Yes or no? Did you get an idea of what data can be collected and for what purpose?

Booth: May 30, 2022, 11:26 AM, Survey "Mummy-Explorer-2"

Number of participants evaluated: 18 (all participants)

Created with LamaPoll | https://www.lamapoll.de

Status data

from 18 Participant	Quantity	Percent
Question seen	18	100.00%
Question answered	18	100.00%
Question not answered	0	0.00%

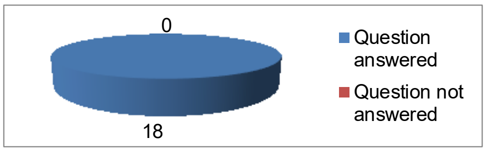

Results

Options	Quantity	Frequency
Yes	18	100.00%
No	0	0.00%
**Total**	18 Answer	18 Participant

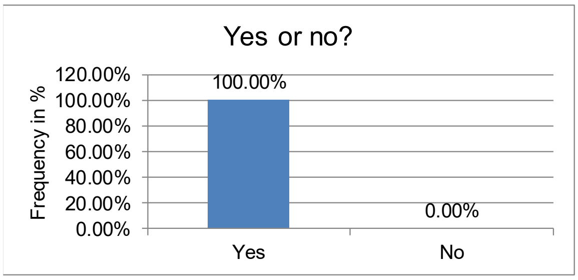

Question 3 - Yes or no? Does the tool contain enough pictures and schemes?

Booth: May 30, 2022, 11:26 AM, Survey "Mummy-Explorer-2"

Number of participants evaluated: 18 (all participants)

Created with LamaPoll | https://www.lamapoll.de

Status data

from 18 Participant	Quantity	Percent
Question seen	18	100.00%
Question answered	18	100.00%
Question not answered	0	0.00%

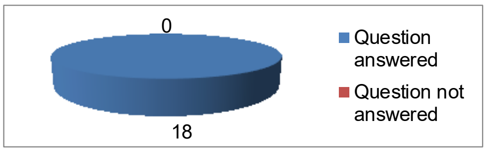

Results

Options	Quantity	Frequency
Yes	14	77.78%
No	4	22.22%
**Total**	18 Answer	18 Participant

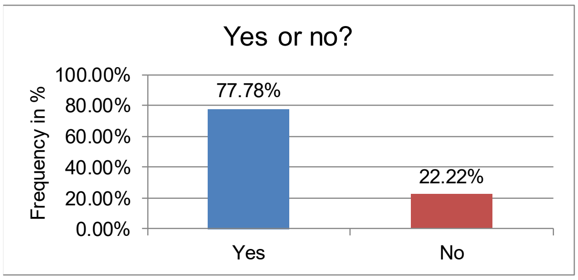

See note of round one

Question 4 - Yes or no? Did you get an idea of how the different research areas are linked?

Booth: May 30, 2022, 11:26 AM, Survey "Mummy-Explorer-2"

Number of participants evaluated: 18 (all participants)

Created with LamaPoll | https://www.lamapoll.de

Status data

from 18 Participant	Quantity	Percent
Question seen	18	100.00%
Question answered	18	100.00%
Question not answered	0	0.00%

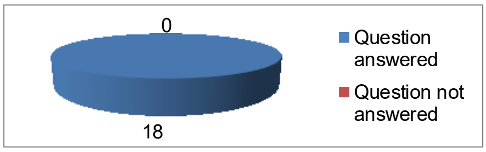

Results

Options	Quantity	Frequency
Yes	15	83.33%
No	3	16.67%
**Total**	18 Answer	18 Participant

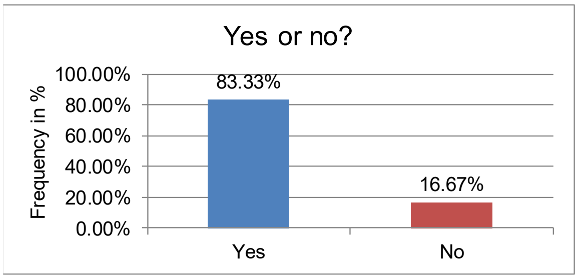

See note of round one.

Question 5 - Yes or no? Are enough references for further reading provided?

Booth: May 30, 2022, 11:26 AM, Survey "Mummy-Explorer-2"

Number of participants evaluated: 18 (all participants)

Created with LamaPoll | https://www.lamapoll.de

Status data

from 18 Participant	Quantity	Percent
Question seen	18	100.00%
Question answered	18	100.00%
Question not answered	0	0.00%

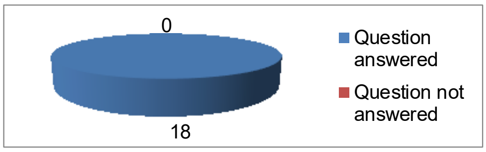

Results

Options	Quantity	Frequency
Yes	16	88.89%
No	2	11.11%
**Total**	18 Answer	18 Participant

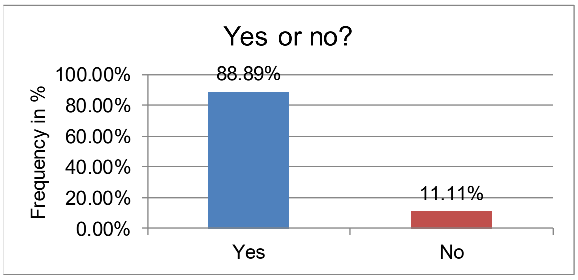

Question 6 - Please fill in the answer. Is there something you did not understand? Why?

Booth: May 30, 2022, 11:26 AM, Survey "Mummy-Explorer-2"

Number of participants evaluated: 18 (all participants)

Created with LamaPoll | https://www.lamapoll.de

Status data

from 18 Participant	Quantity	Percent
Question seen	18	100.00%
Question answered	6	33.33%
Question not answered	12	66.67%

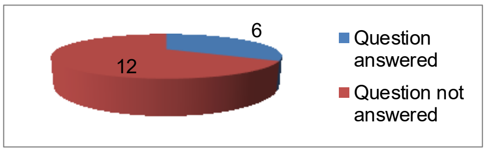

Result details for Please fill in the answer

Quantity Answers	6	Quantity clear	6

For data protection reasons individual answers are not shown but students confirmed that they understood the content.

Question 7 - Yes or no? Could you open the tool?

Booth: May 30, 2022, 11:26 AM, Survey "Mummy-Explorer-2"

Number of participants evaluated: 18 (all participants)

Created with LamaPoll | https://www.lamapoll.de

Status data

from 18 Participant	Quantity	Percent
Question seen	18	100.00%
Question answered	18	100.00%
Question not answered	0	0.00%

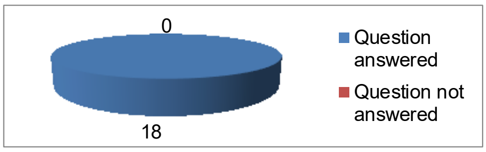

Results

Options	Quantity	Frequency
Yes	18	100.00%
No	0	0.00%
**Total**	18 Answer	18 Participant

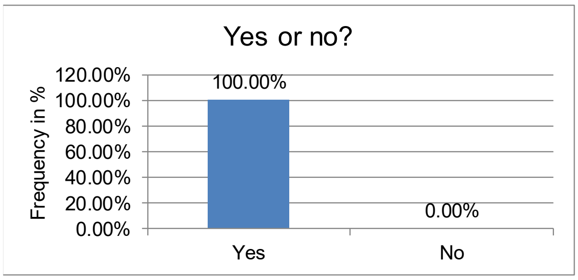

Question 8 - Yes or no? Do the pages load fast enough?

Booth: May 30, 2022, 11:26 AM, Survey "Mummy-Explorer-2"

Number of participants evaluated: 18 (all participants)

Created with LamaPoll | https://www.lamapoll.de

Status data

from 18 Participant	Quantity	Percent
Question seen	18	100.00%
Question answered	18	100.00%
Question not answered	0	0.00%

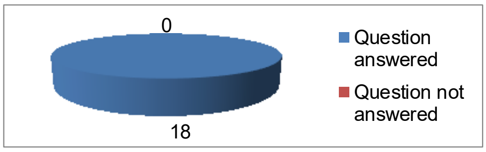

Results

Options	Quantity	Frequency
Yes	14	77.78%
No	4	22.22%
**Total**	18 Answer	18 Participant

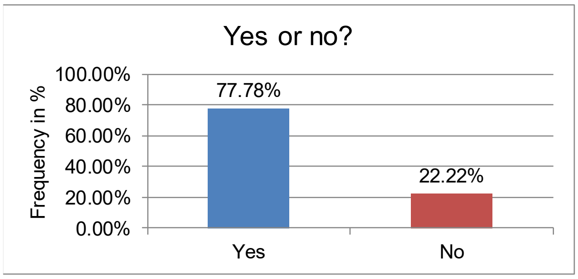

See note of round one.

Question 9 - Please fill in the answer. Did you have any other technical difficulties?

Booth: May 30, 2022, 11:26 AM, Survey "Mummy-Explorer-2"

Number of participants evaluated: 18 (all participants)

Created with LamaPoll | https://www.lamapoll.de

Status data

from 18 Participant	Quantity	Percent
Question seen	18	100.00%
Question answered	11	61.11%
Question not answered	7	38.89%

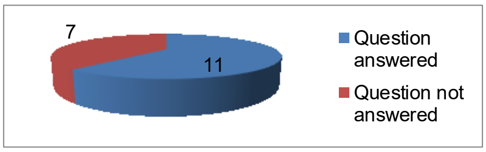

Result details for Please fill in the answer

Quantity Answers	11	Quantity clear	9

See note of round one.

Question 10 - Yes or no? Is the content of the pictures visible and the resolution good enough?

Booth: May 30, 2022, 11:26 AM, Survey "Mummy-Explorer-2"

Number of participants evaluated: 18 (all participants)

Created with LamaPoll | https://www.lamapoll.de

Status data

from 18 Participant	Quantity	Percent
Question seen	18	100.00%
Question answered	18	100.00%
Question not answered	0	0.00%

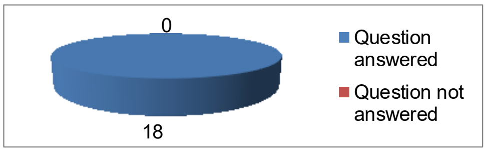

Results

Options	Quantity	Frequency
Yes	18	100.00%
No	0	0.00%
**Total**	18 Answer	18 Participant

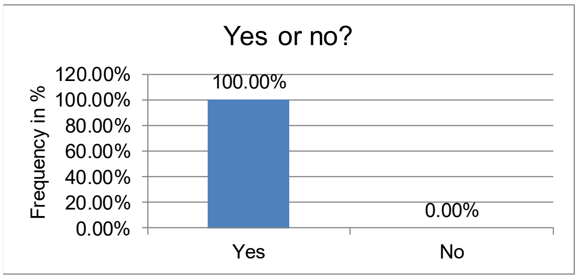

Question 11 - Please fill in the answer. Space for more comments. What did you like? What should be improved?

Booth: May 30, 2022, 11:26 AM, Survey "Mummy-Explorer-2"

Number of participants evaluated: 18 (all participants)

Created with LamaPoll | https://www.lamapoll.de

Status data

from 18 Participant	Quantity	Percent
Question seen	18	100.00%
Question answered	13	72.22%
Question not answered	5	27.78%

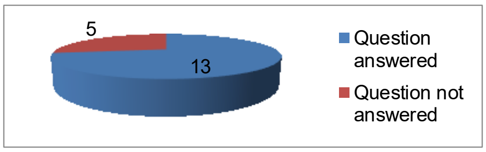

Result details for Please fill in the answer

Quantity Answers	13	Quantity clear	13

See note of round one.

SI Note III Results of the Lecturers Questionnaire

Note: After the survey more details were added to the manual.

Participation overview

	Quantity	Quote
Visitors	29	-
Participations	15	51.72%
Returns	13	86.67%
Dropout	2	13.33%

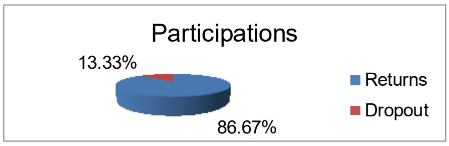

Participant	launched	ends	aborted
Anonymous	15	13	2
Access key	0	0	0
Address book	0	0	0
**Total**	**15**	**13**	**2**

Question 1 - Yes or no? Could you download the software?

Booth: May 30, 2022, 11:26 AM, Survey "Docent-part"

Number of participants evaluated: 15 (all participants)

Created with LamaPoll | https://www.lamapoll.de

Status data

from 15 Participant	Quantity	Percent
Question seen	15	100.00%
Question answered	15	100.00%
Question not answered	0	0.00%

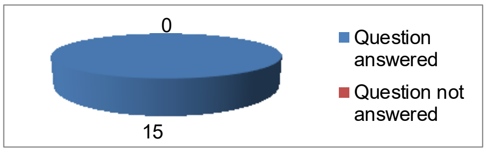

Results

Options	Quantity	Frequency
Yes	14	93.33%
No	1	6.67%
**Total**	15 Answer	15 Participant

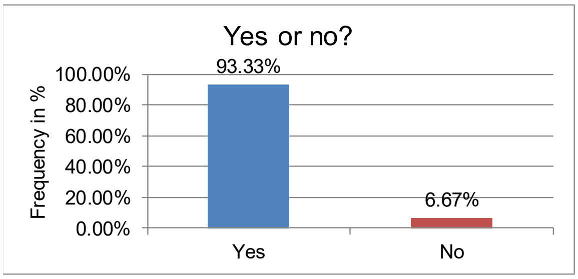

Question 2 - Yes or no? Could you download the template and open it in the Google Web Designer?

Booth: May 30, 2022, 11:26 AM, Survey "Docent-part"

Number of participants evaluated: 15 (all participants)

Created with LamaPoll | https://www.lamapoll.de

Status data

from 15 Participant	Quantity	Percent
Question seen	15	100.00%
Question answered	13	86.67%
Question not answered	2	13.33%

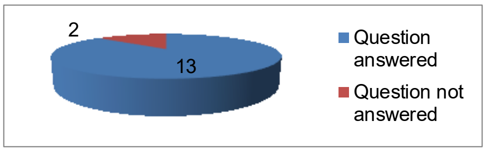

Results

Options	Quantity	Frequency
Yes	11	84.62%
No	2	15.38%
**Total**	13 Answer	13 Participant

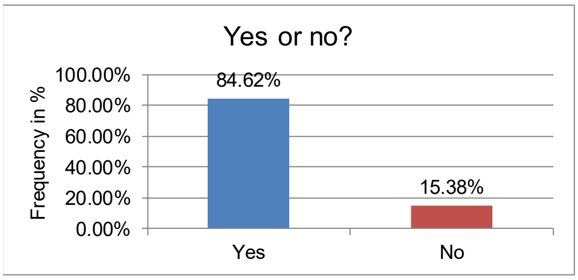

Question 3 - Yes or no? Are the steps in the manual sufficiently explained?

Booth: May 30, 2022, 11:26 AM, Survey "Docent-part"

Number of participants evaluated: 15 (all participants)

Created with LamaPoll | https://www.lamapoll.de

Status data

from 15 Participant	Quantity	Percent
Question seen	14	93.33%
Question answered	13	86.67%
Question not answered	2	13.33%

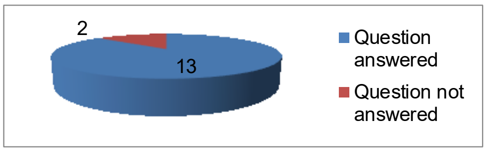

Results

Options	Quantity	Frequency
Yes	5	38.46%
No	8	61.54%
**Total**	13 Answer	13 Participant

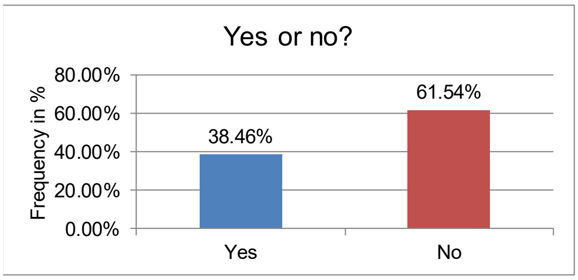

Question 4 - Please fill in the answer. Which steps need more details?

Booth: May 30, 2022, 11:26 AM, Survey "Docent-part"

Number of participants evaluated: 15 (all participants)

Created with LamaPoll | https://www.lamapoll.de

Status data

from 15 Participant	Quantity	Percent
Question seen	14	93.33%
Question answered	12	80.00%
Question not answered	3	20.00%

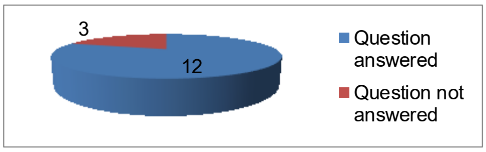

Result details for Please fill in the answer

Quantity Answers	12	Quantity clear	12

For data protection reasons individual answers are not shown.

Question 5 - Please fill in the answer. What problems did you experience when setting up your own version?

Booth: May 30, 2022, 11:26 AM, Survey "Docent-part"

Number of participants evaluated: 15 (all participants)

Created with LamaPoll | https://www.lamapoll.de

Status data

from 15 Participant	Quantity	Percent
Question seen	13	86.67%
Question answered	12	80.00%
Question not answered	3	20.00%

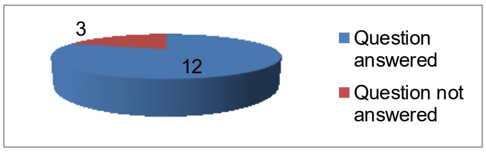

Result details for Please fill in the answer

Quantity Answers	12	Quantity clear	12

For data protection reasons individual answers are not shown.

Question 6 - Yes or no? Do you think providing a template is useful and can you think of other lectures where such a tool could be useful?

Booth: May 30, 2022, 11:26 AM, Survey "Docent-part"

Number of participants evaluated: 15 (all participants)

Created with LamaPoll | https://www.lamapoll.de

Status data

from 15 Participant	Quantity	Percent
Question seen	13	86.67%
Question answered	13	86.67%
Question not answered	2	13.33%

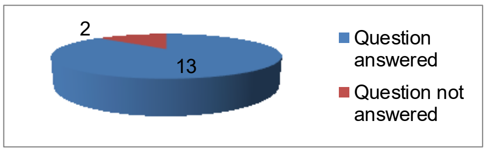

Results

Options	Quantity	Frequency
Yes	12	92.31%
No	1	7.69%
**Total**	13 Answer	13 Participant

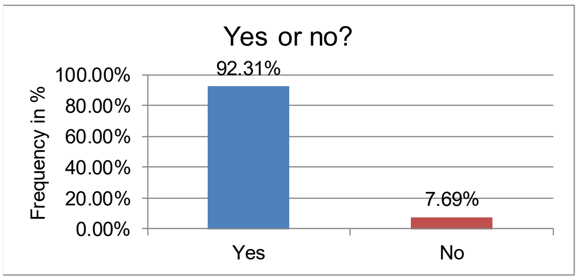

SI Note IV Results of the Second Student Questionnaire

Survey "Improved-student-survey"

Booth: Jan 10, 2023, 02:33 PM

Number of participants evaluated: 13 (all participants)

Created with LamaPoll | https://www.lamapoll.de

Participation overview

	Quantity	Quote
Visitors	52	-
Participations	39	75.00%
Returns	32	61.54%
Dropout	7	13.46%

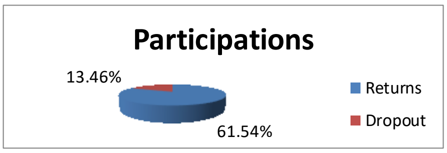

Participant	launched	ends	aborted
Anonymous	39	32	7
Access key	0	0	0
Address book	0	0	0
**Total**	**13**	**12**	**1**

Question 1 - Could you open the tool?

Booth: Dec 22, 2022, 05:15 PM, Survey "Improved-student-survey"

Number of participants evaluated: 26 (all participants)

Created with LamaPoll | https://www.lamapoll.de

Status data

from 39 Participant	Quantity	Percent
Question seen	38	100.00%
Question answered	34	89.47%
Question not answered	4	10.53%

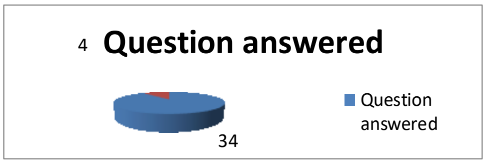

Results (Total)

Frequency in %	Value 1	Value 2	Value 3	Value 4	Value 5	Total
Could you open the tool?	0.00%	0.00%	0.00%	8.82%	91.18%	34

Frequency Quantity	Value 1	Value 2	Value 3	Value 4	Value 5	Total	Mean value	Median
Could you open the tool?	0	0	0	3	31	34	4.91	5
**Total**				3	31	**34**	**4.91**	**5**

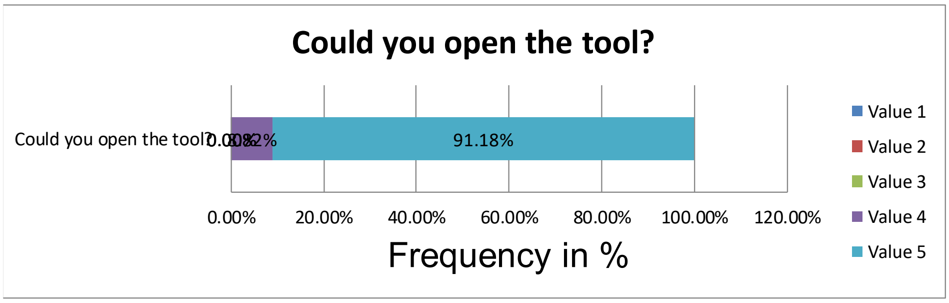

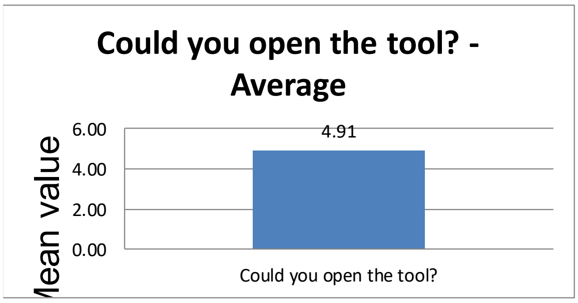

Question 2 - Does the tool provide an overview of possible analysis for the study of mummies?

Booth: Dec 22, 2022, 05:15 PM, Survey "Improved-student-survey"

Number of participants evaluated: 26 (all participants)

Created with LamaPoll | https://www.lamapoll.de

Status data

from 39 Participant	Quantity	Percent
Question seen	38	100.00%
Question answered	30	78.95%
Question not answered	8	21.05%

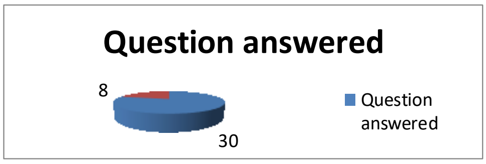

Results (Total)

Frequency in %	Value 1	Value 2	Value 3	Value 4	Value 5	Total
Does the tool provide an overvie	0.00%	0.00%	3.33%	36.67%	60.00%	30

Frequency Quantity	Value 1	Value 2	Value 3	Value 4	Value 5	Total	Mean value	Median
Does the tool provide an overvie	0	0	1	11	18	30	4.56	5
**Total**			1	11	18	**30**	**4.56**	**5**

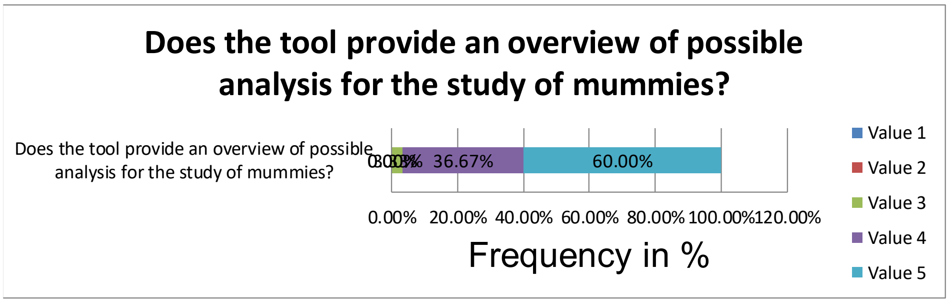

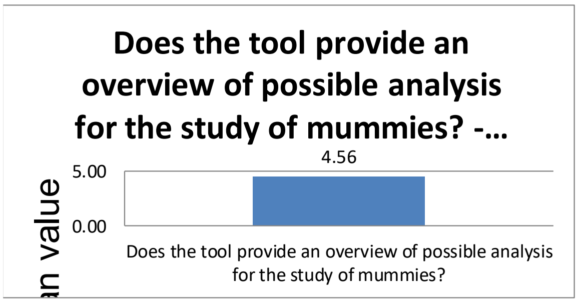

Question 3 - Did you get an idea of what data can be collected and for what purpose?

Booth: Dec 22, 2022, 05:15 PM, Survey "Improved-student-survey"

Number of participants evaluated: 26 (all participants)

Created with LamaPoll | https://www.lamapoll.de

Status data

from 39 Participant	Quantity	Percent
Question seen	34	84.62%
Question answered	28	82.35%
Question not answered	10	29.41%

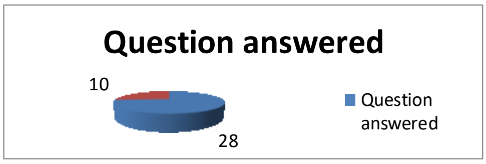

Results (Total)

Frequency in %	Value 1	Value 2	Value 3	Value 4	Value 5	Total
Did you get an idea of what data	0.00%	3.57%	0.00%	28.57%	67.86%	28

Frequency Quantity	Value 1	Value 2	Value 3	Value 4	Value 5	Total	Mean value	Median
Did you get an idea of what data	0	1	0	8	19	28	4.60	5
**Total**		1		8	19	**28**	**4.60**	**5**

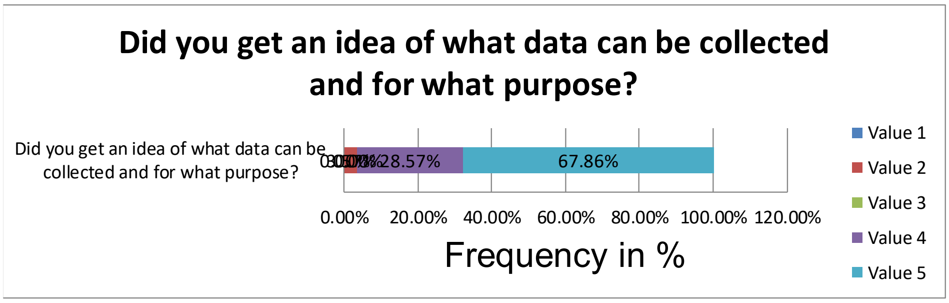

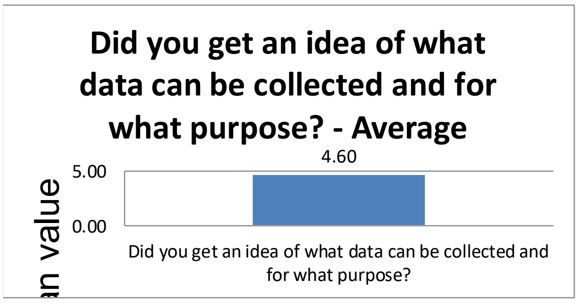

Question 4 - Does the tool contain enough pictures and schemes?

Booth: Dec 22, 2022, 05:15 PM, Survey "Improved-student-survey"

Number of participants evaluated: 26 (all participants)

Created with LamaPoll | https://www.lamapoll.de

Status data

from 39 Participant	Quantity	Percent
Question seen	34	84.62%
Question answered	26	76.47%
Question not answered	12	35.29%

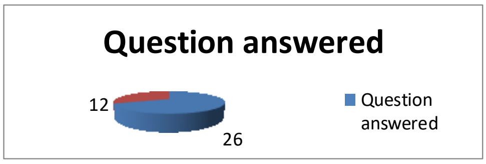

Results (Total)

Frequency in %	Value 1	Value 2	Value 3	Value 4	Value 5	Total
Does the tool contain enough pi	15.38%	11.54%	11.54%	23.08%	38.46%	26

Frequency Quantity	Value 1	Value 2	Value 3	Value 4	Value 5	Total	Mean value	Median
Does the tool contain enough pi	4	3	3	6	10	26	3.58	4
**Total**	**4**	**3**	**3**	**6**	**10**	**26**	**3.58**	**4**

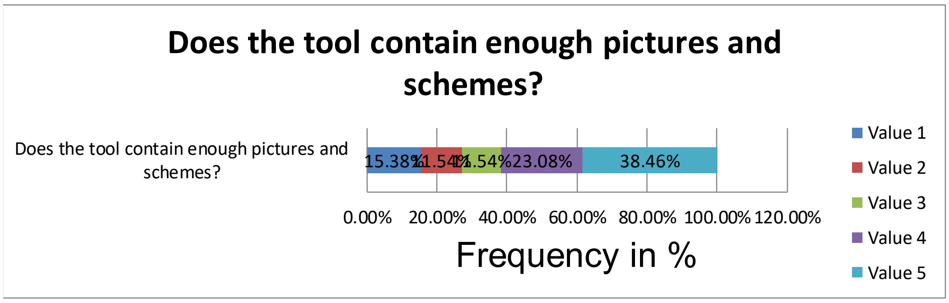

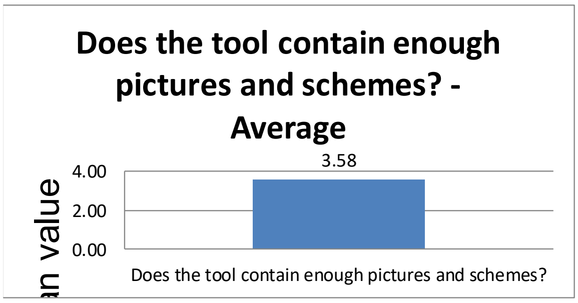

Question 5 - Did you get an idea of how the different research areas are linked?

Booth: Dec 22, 2022, 05:15 PM, Survey "Improved-student-survey"

Number of participants evaluated: 26 (all participants)

Created with LamaPoll | https://www.lamapoll.de

Status data

from 39 Participant	Quantity	Percent
Question seen	33	84.62%
Question answered	28	65.38%
Question not answered	10	34.62%

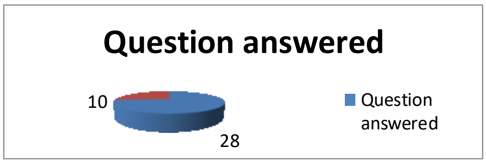

Results (Total)

Frequency in %	Value 1	Value 2	Value 3	Value 4	Value 5	Total
Did you get an idea of how the d	0.00%	7.14%	10.71%	42.86%	39.29%	28

Frequency Quantity	Value 1	Value 2	Value 3	Value 4	Value 5	Total	Mean value	Median
Did you get an idea of how the d	0	2	3	12	11	28	4.14	4
**Total**		2	3	12	11	**28**	**4.14**	**4**

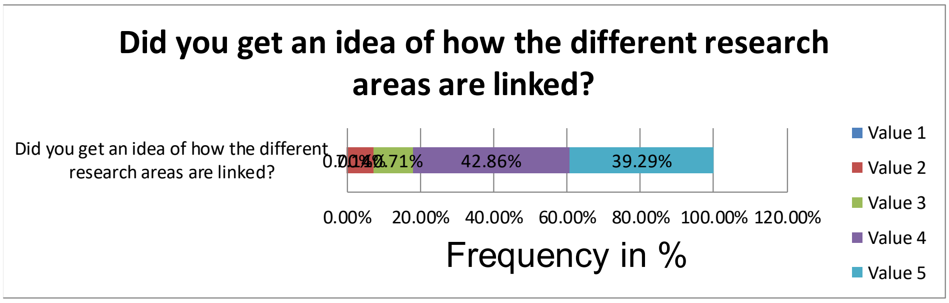

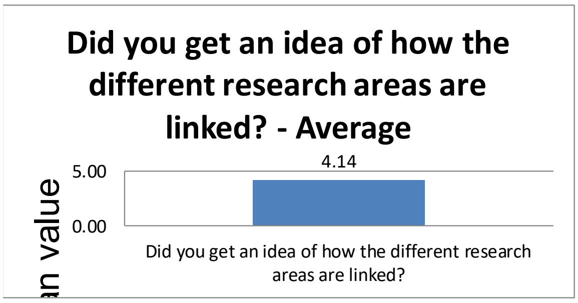

Question 6 - Are enough references for further reading provided?

Booth: Dec 22, 2022, 05:15 PM, Survey "Improved-student-survey"

Number of participants evaluated: 26 (all participants)

Created with LamaPoll | https://www.lamapoll.de

Status data

from 39 Participant	Quantity	Percent
Question seen	33	84.62%
Question answered	28	65.38%
Question not answered	10	34.62%

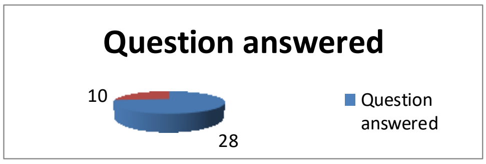

Results (Total)

Frequency in %	Value 1	Value 2	Value 3	Value 4	Value 5	Total
Are enough references for furthe	14.29%	7.14%	3.57%	35.71%	39.29%	28

Frequency Quantity	Value 1	Value 2	Value 3	Value 4	Value 5	Total	Mean value	Median
Are enough references for furthe	4	2	1	10	11	28	3.79	4
**Total**	4	2	1	10	11	**28**	**3.79**	**4**

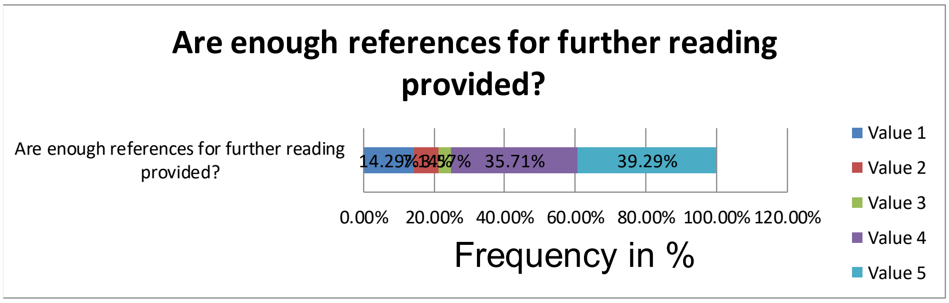

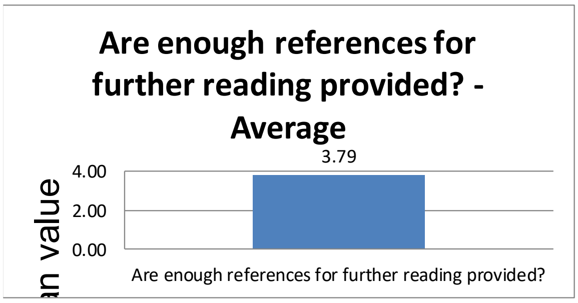

Question 7 - Is there something you did not understand?

Booth: Dec 22, 2022, 05:15 PM, Survey "Improved-student-survey-1"

Number of participants evaluated: 12 (all participants)

Created with LamaPoll | https://www.lamapoll.de

Status data

from 24 Participant	Quantity	Percent
Question seen	22	91.67%
Question answered	22	91.67%
Question not answered	2	8.33%

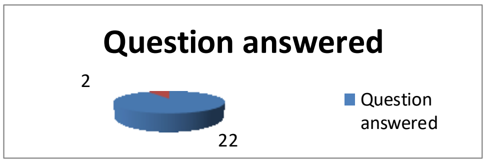

Results

Options	Quantity	Frequency
Yes	2	9.09%
No	20	90.91%
**Total**	11 Answer	11 Participant

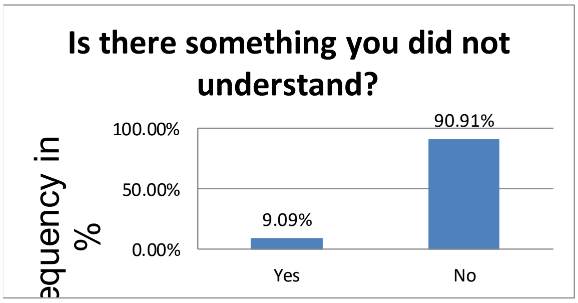

Question 8 - If yes, why?

Booth: Dec 22, 2022, 05:15 PM, Survey "Improved-student-survey"

Number of participants evaluated: 26 (all participants)

Created with LamaPoll | https://www.lamapoll.de

Status data

from 38 Participant	Quantity	Percent
Question seen	33	84.62%
Question answered	4	7.69%
Question not answered	34	92.31%

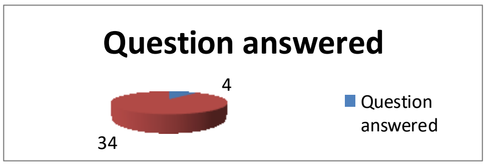

For data protection reasons individual answers are not shown but students confirmed that they understood the content.

Question 9 - Do you think the Mummy Explorer can support your learning experience?

Booth: Dec 22, 2022, 05:15 PM, Survey "Improved-student-survey"

Number of participants evaluated: 26 (all participants)

Created with LamaPoll | https://www.lamapoll.de

Status data

from 38 Participant	Quantity	Percent
Question seen	29	69.23%
Question answered	23	50.00%
Question not answered	15	50.00%

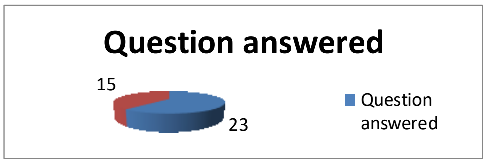

Results (Total)

Frequency in %	Value 1	Value 2	Value 3	Value 4	Value 5	Total
Do you think the Mummy Explor	13.04%	8.70%	0.00%	30.43%	47.83%	23

Frequency Quantity	Value 1	Value 2	Value 3	Value 4	Value 5	Total	Mean value	Median
Do you think the Mummy Explor	3	2	0	7	11	23	3.91	4
**Total**	3	2		7	11	**23**	**3.91**	**4**

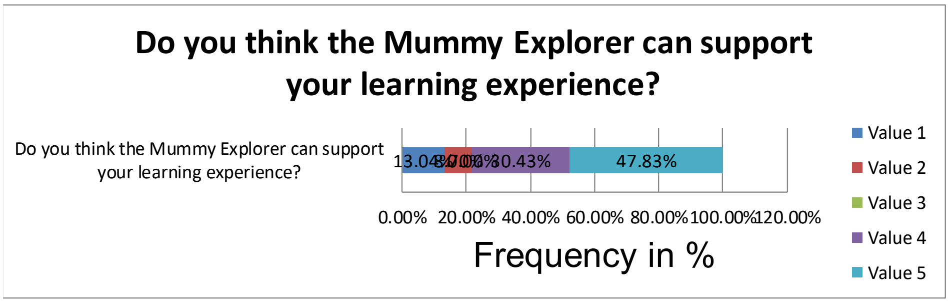

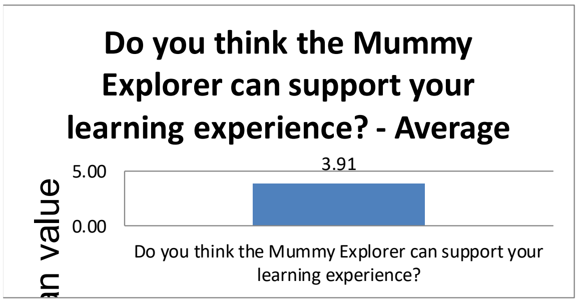

Question 10 - Do the pages load fast enough?

Booth: Dec 22, 2022, 05:15 PM, Survey "Improved-student-survey"

`Number of participants evaluated: 26 (all participants)`

`Created with LamaPoll | https://www.lamapoll.de`

Status data

from 38 Participant	Quantity	Percent
Question seen	32	84.21%
Question answered	31	81.58%
Question not answered	7	18.42%

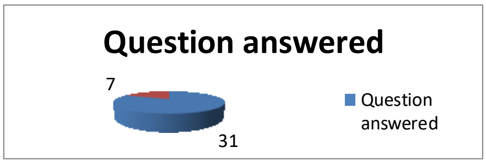

Results (Total)

Frequency in %	Value 1	Value 2	Value 3	Value 4	Value 5	Total
Do the pages load fast enough?	3.23%	3.23%	3.23%	9.68%	80.65%	31

Frequency Quantity	Value 1	Value 2	Value 3	Value 4	Value 5	Total	Mean value	Median
Do the pages load fast enough?	1	1	1	3	25	31	4.61	5
**Total**	1	1	1	3	25	**31**	**4.61**	**5**

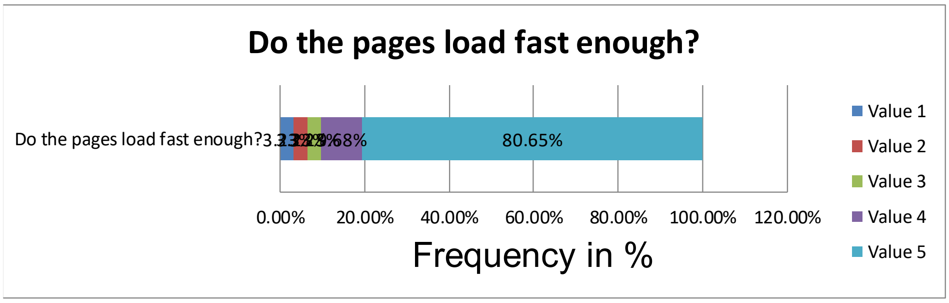

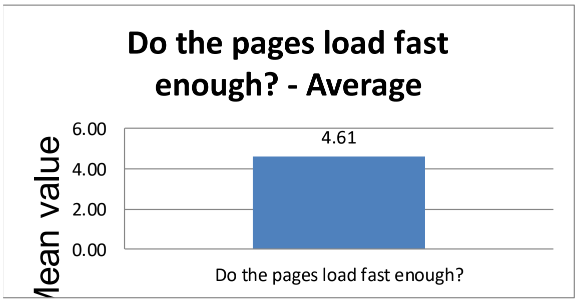

Question 11 - Did you have any other technical difficulties?

Booth: Dec 22, 2022, 05:15 PM, Survey "Improved-student-survey"

`Number of participants evaluated: 26 (all participants)`

`Created with LamaPoll | https://www.lamapoll.de`

Status data

from 38 Participant	Quantity	Percent
Question seen	32	84.21%
Question answered	10	26.32%
Question not answered	27	71.05%

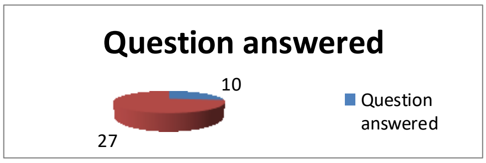

For data protection reasons individual answers are not shown but 6 out of the 10 who answered, answered with No.

Question 12 - Is the content of the pictures visible and the resolution good enough?

Booth: Dec 22, 2022, 05:15 PM, Survey "Improved-student-survey"

Number of participants evaluated: 26 (all participants)

Created with LamaPoll | https://www.lamapoll.de

Status data

from 38 Participant	Quantity	Percent
Question seen	31	81.58%
Question answered	28	73.68%
Question not answered	10	26.32%

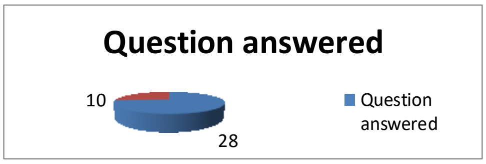

Results (Total)

Frequency in %	Value 1	Value 2	Value 3	Value 4	Value 5	Total
Is the content of the pictures visi	3.57%	10.71%	3.57%	32.14%	50.00%	28

Frequency Quantity	Value 1	Value 2	Value 3	Value 4	Value 5	Total	Mean value	Median
Is the content of the pictures visi	1	3	1	9	14	28	4.41	5
**Total**	1	3	1	9	14	**28**	**4.41**	**5**

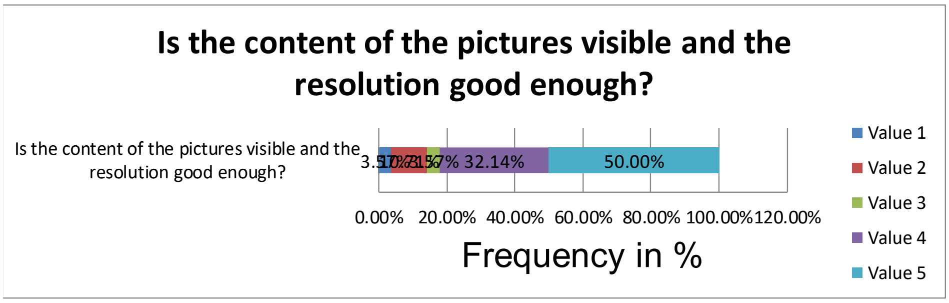

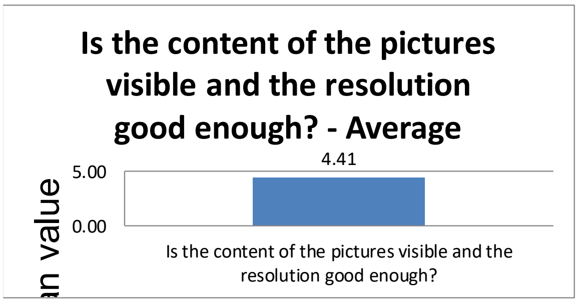

Question 13 - Space for more comments. What did you like? What should be improved?

Booth: Dec 22, 2022, 05:15 PM, Survey "Improved-student-survey"

Number of participants evaluated: 26 (all participants)

Created with LamaPoll | https://www.lamapoll.de

Status data

from 38 Participant	Quantity	Percent
Question seen	32	84.21%
Question answered	14	36.84%
Question not answered	24	63.16%

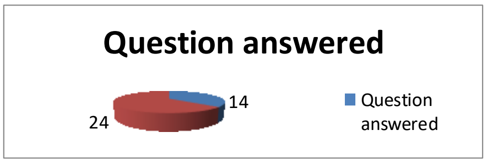

For data protection reasons individual answers are not shown. But points that were raised: the design of the tools still looks outdated and the tool is not compatible with mobile devices.
